# phydms: software for phylogenetic analyses informed by deep mutational scanning

**DOI:** 10.7717/peerj.3657

**Published:** 2017-07-31

**Authors:** Sarah K. Hilton, Michael B. Doud, Jesse D. Bloom

**Affiliations:** 1 Division of Basic Sciences and Computational Biology Program, Fred Hutchinson Cancer Research Center, Seattle, WA, USA; 2 Department of Genome Sciences, University of Washington, Seattle, WA, United States of America; 3 Medical Scientist Training Program, University of Washington, Seattle, WA, United States of America

**Keywords:** Deep mutational scanning, Phylogenetics, ExpCM, Codon substitution model, Diversifying selection, Positive selection, dN/dS, Hemagglutinin, Beta lactamase, Amino acid preferences

## Abstract

It has recently become possible to experimentally measure the effects of all amino-acid point mutations to proteins using deep mutational scanning. These experimental measurements can inform site-specific phylogenetic substitution models of gene evolution in nature. Here we describe software that efficiently performs analyses with such substitution models. This software, phydms, can be used to compare the results of deep mutational scanning experiments to the selection on genes in nature. Given a phylogenetic tree topology inferred with another program, phydms enables rigorous comparison of how well different experiments on the same gene capture actual natural selection. It also enables re-scaling of deep mutational scanning data to account for differences in the stringency of selection in the lab and nature. Finally, phydms can identify sites that are evolving differently in nature than expected from experiments in the lab. As data from deep mutational scanning experiments become increasingly widespread, phydms will facilitate quantitative comparison of the experimental results to the actual selection pressures shaping evolution in nature.

## Introduction

It is widely appreciated that experiments in the lab can inform understanding of protein evolution in nature (Dean & Thornton, 2007; Harms & Thornton, 2013). Efforts to synthesize experiments with evolutionary data have typically involved generating protein variants of interest, assaying their functionality in the lab, and qualitatively comparing the measured functionality of each variant to its evolutionary fate in nature (Dean & Thornton, 2007; Harms & Thornton, 2013). The recent advent of high-throughput deep mutational scanning techniques (Fowler & Fields, 2014) has greatly expanded the potential of such research. For instance, numerous recent papers have reported measuring the effects of *all* amino-acid mutations on the functionality of a range of proteins (McLaughlin et al., 2012; Roscoe et al., 2013; Firnberg et al., 2014; Olson, Wu & Sun, 2014; Melnikov et al., 2014; Bloom, 2014a; Thyagarajan & Bloom, 2014; Stiffler, Hekstra1 & Ranganathan, 2015; Doud, Ashenberg & Bloom, 2015; Kitzman et al., 2015; Mishra et al., 2016; Doud & Bloom, 2016; Mavor et al., 2016; Haddox, Dingens & Bloom, 2016; Fernandes et al., 2016; Majithia et al., 2016; Brenan et al., 2016). This flood of data necessitates new methods for comparing experimental measurements to evolution in nature, since simple qualitative inspection is insufficient when measurements are available for tens of thousands of mutants.

A solution is provided by the methods of molecular phylogenetics. Longstanding phylogenetic algorithms enable calculation of the statistical likelihood of an alignment of naturally occurring gene sequences given a phylogenetic tree and a model for the evolutionary substitution process (Felsenstein, 1973; Felsenstein, 1981). Deep mutational scanning data can be incorporated into this statistical framework via the substitution model (Bloom, 2014a). Such an experimentally informed codon model (ExpCM) of substitution can be used to test whether a deep mutational scanning experiment provides evolutionarily relevant information (Bloom, 2014a), compare the stringency of selection in nature and the lab (Bloom, 2014b), assess how well different experiments describe natural selection on the same gene (Doud, Ashenberg & Bloom, 2015), and identify sites that are evolving differently in nature than expected from experiments in the lab (Bloom, 2017).

However, a hindrance to such analyses has been the lack of appropriate software. Prior work using an ExpCM has re-purposed existing software such as HyPhy (Pond, Frost & Muse, 2005) or Bio++ (Guéguen et al., 2013) to optimize the phylogenetic likelihood. Because these existing software packages are not designed for such site-specific models, the resulting analyses have been slow and cumbersome. Other software packages (Tamuri, Dos Reis & Goldstein, 2012; Tamuri, Goldman & Dos Reis, 2014; Rodrigue, Philippe & Lartillot, 2010; Rodrigue & Lartillot, 2014) that handle site-specific codon substitution models are designed to treat the effects of mutations as unknowns to be inferred rather than as values that have been measured *a priori*.

Here we describe phydms, software for **phy**logenetics informed by **d**eep **m**utational **s**canning. We show that phydms is ∼100-fold faster than existing alternatives for performing analyses with an ExpCM, and demonstrate how it can be used to quantitatively relate measurements from deep mutational scanning with selection in nature. Readers who are interested in technical details of how phydms works should read the METHODS section; readers who are primarily interested in simply using phydms may prefer to jump directly to the ‘RESULTS’ section.

## Methods

### Substitution models

#### Experimentally informed codon model (ExpCM)

The basic ExpCM implemented in phydms is identical to those in Bloom (2017). We recap this ExpCM to introduce nomenclature needed to understand the extensions described in the next few subsections.

In an ExpCM, rate of substitution *P*_*r*,*xy*_ of site *r* from codon *x* to *y* is written in mutation-selection form Halpern & Bruno (1998), McCandlish & Stoltzfus (2014) and Spielman & Wilke (2015) as

(1) \begin{eqnarray*}{P}_{r,xy}={Q}_{xy}\times {F}_{r,xy}\end{eqnarray*}

where *Q*_*xy*_ is proportional to the rate of mutation from *x* to *y*, and *F*_*r*,*xy*_ is proportional to the probability that this mutation fixes. The rate of mutation *Q*_*xy*_ is assumed to be uniform across sites, and takes an HKY85-like (Hasegawa, Kishino & Yano, 1985) form:

(2) \begin{eqnarray*}{Q}_{xy}= \left\{ \begin{array}{@{}ll@{}} \displaystyle {\phi }_{w} &\displaystyle \text{if} x \text{and} y \text{differ by a transversion to nucleotide} w\\ \displaystyle \kappa {\phi }_{w} &\displaystyle \text{if} x \text{and} y \text{differ by a transition to nucleotide} w\\ \displaystyle 0 &\displaystyle \text{if} x \text{and} y \text{differ by}\gt 1 \text{nucleotide}. \end{array} \right. \end{eqnarray*}

The *κ* parameter represents the transition-transversion ratio, and the *ϕ*_*w*_ values give the expected frequency of nucleotide *w* in the absence of selection on amino-acid substitutions, and are constrained by 1 = ∑_*w*_*ϕ*_*w*_.

The deep mutational scanning data are incorporated into the ExpCM via the *F*_*r*,*xy*_ terms. The experiments measure the preference *π*_*r*,*a*_ of every site *r* for every amino-acid *a* (see the ‘RESULTS’ section for more details on these preferences). The *F*_*r*,*xy*_ terms are defined in terms of these experimentally measured amino-acid preferences as

**Figure 1:**
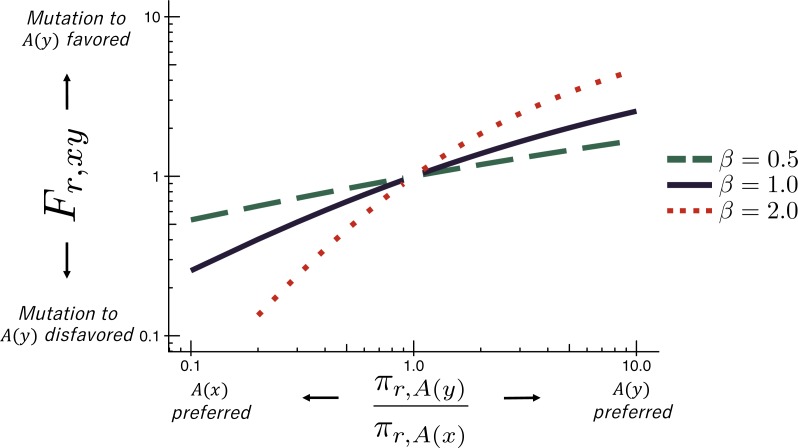
The ExpCM fixation term *F*_*r*,*xy*_. In an ExpCM, the rate of fixation of a mutation from codon *x* to codon *y* depends on the experimentally measured preferences of the amino acids $A \left( x \right) $ and $A \left( y \right) $ encoded by these codons. Mutations to preferred amino acids, with $ \frac{{\pi }_{r,A \left( y \right) }}{{\pi }_{r,A \left( x \right) }} \gt 1$, result in a larger *F*_*r*,*xy*_, and so are anticipated to fix more often. The value of *F*_*r*,*xy*_ is modulated by re-scaling the preferences by a stringency parameter *β* ≠ 1 to reflect differences in selection between the lab and nature. When *β* > 1, the selection for preferred amino acids is exaggerated. When *β* < 1, the selection for preferred amino acids is attenuated.

(3) \begin{eqnarray*}{F}_{r,xy}= \left\{ \begin{array}{@{}ll@{}} \displaystyle 1 &\displaystyle \text{if} \mathcal{A} \left( x \right) =\mathcal{A} \left( y \right) \text{}\\ \displaystyle \omega \times \frac{\ln \nolimits \left[ { \left( {\pi }_{r,\mathcal{A} \left( y \right) }/{\pi }_{r,\mathcal{A} \left( x \right) } \right) }^{\beta } \right] }{1-{ \left( {\pi }_{r,\mathcal{A} \left( x \right) }/{\pi }_{r,\mathcal{A} \left( y \right) } \right) }^{\beta }} &\displaystyle \text{if} \mathcal{A} \left( x \right) \not = \mathcal{A} \left( y \right) \text{} \end{array} \right. \end{eqnarray*}

where $\mathcal{A} \left( x \right) $ is the amino-acid encoded by codon *x*, *β* is the stringency parameter, and *ω* is the relative rate of nonsynonymous to synonymous substitutions after accounting for the amino-acid preferences. As shown in Fig. 1, Eq. (3) implies that mutations to more preferred amino acids are favored, and mutations to less preferred amino acids are disfavored. The functional form in Eq. (3) was derived by Halpern & Bruno (1998) and under certain (probably unrealistic) population-genetic assumptions; under these assumptions, *β* is related to the effective population size. When *β* > 1, natural evolution favors the same mutations as the experiments but with greater stringency. The ExpCM has six free parameters (three *ϕ*_*w*_ values, *κ*, *β*, and *ω*). The preferences *π*_*r*,*a*_ are *not* free parameters since they are determined by an experiment independent of the sequence alignment being analyzed.

#### ExpCM with empirical nucleotide frequency parameters

Phylogenetic substitution models commonly set the nucleotide frequency parameters (*ϕ*_*w*_ in the case of an ExpCM) so that the model’s stationary state equals the empirical frequencies of the characters in the alignment. Setting the frequency parameters in this way reduces the number of parameters that must be optimized by maximum likelihood. Empirically setting the nucleotide frequency parameters is easy for substitution models where the stationary state only depends on these parameters.

However, the situation for an ExpCM is more complex. The *ϕ*_*w*_ values give the expected nucleotide frequencies in the *absence* of selection on amino acids, but in an ExpCM there is site-specific selection on amino acids. Therefore, the stationary state of an ExpCM also depends on other quantities: the stationary state frequency *p*_*r*,*x*_ of codon *x* at site *r* is (Bloom, 2017)

(4) \begin{eqnarray*}{p}_{r,x}= \frac{{ \left( {\pi }_{r,\mathcal{A} \left( x \right) } \right) }^{\beta }{\phi }_{{x}_{0}}{\phi }_{{x}_{1}}{\phi }_{{x}_{2}}}{\sum _{z}{ \left( {\pi }_{r,\mathcal{A} \left( z \right) } \right) }^{\beta }{\phi }_{{z}_{0}}{\phi }_{{z}_{1}}{\phi }_{{z}_{2}}} ,\end{eqnarray*}

where *x*_*k*_ indicates the nucleotide at position *k* in codon *x*. As this equation makes clear, the stationary state of an ExpCM depends on the preferences *π*_*r*,*a*_ and stringency parameter *β* as well as the nucleotide frequency parameters *ϕ*_*w*_.

So for an ExpCM, setting *ϕ*_*w*_ empirically means choosing their values such that the alignment frequency *g*_*w*_ of nucleotide *w* is as expected given the stationary state *p*_*r*,*x*_. This will be the case if the following equation holds for all *w*:

(5) \begin{eqnarray*}{g}_{w}= \frac{1}{L} \sum _{r}\sum _{x} \frac{1}{3} {N}_{w} \left( x \right) {p}_{r,x}\end{eqnarray*}

where *L* is the length of the gene in codons, *r* ranges over all codon sites, *x* ranges over all codon identities, and ${N}_{w} \left( x \right) $ is the number of occurrences of nucleotide *w* in codon *x*. We could not analytically solve this system of equations for *ϕ*_*w*_ in terms of *g*_*w*_, so we instead used a non-linear equation solver to determine the values as detailed in File S1. Calculating *ϕ*_*w*_ empirically in this fashion is the default for phydms. If you instead want to fit the *ϕ*_*w*_ values, use the –fitphi option.

#### ExpCM with gamma-distributed nonsynonymous-to-synonymous rate parameter

A common extension to traditional non-site-specific codon substitution models is to allow the dN/dS ratio *ω* to come from several discrete categories by making the overall likelihood at each site a linear combination of the likelihood computed for each category (Nielsen & Yang, 1998; Yang et al., 2000). Such models are not site-specific since sites are not assigned to a category during likelihood optimization, but they do capture the idea that the strength of selection on nonsynonymous mutations varies across sites.

One variant of this approach draws *ω* from a discrete gamma distribution. This variant is referred to as the M5 variant (Yang et al., 2000) in PAML (Yang, 2007). We implemented a similar approach for an ExpCM, following Yang (1994) to draw the *ω* in Eq. (3) from the means of equally weighted gamma-distributed categories (by default there are four categories). This option can be used via the –gammaomega switch to phydms, and adds one free parameter, since there are two parameters controlling the gamma distribution (a shape and inverse-scale parameter) rather than a single *ω*. This option increases the runtime by ∼5-fold.

Using a gamma-distributed *ω* may lead to less of an improvement in fit for an ExpCM than for non-site-specific models, since much of the site-to-site variation in the selection is already captured by the amino-acid preferences. However, it can still lead to substantial improvements if a subset of sites are under diversifying selection or if the preferences do not fully capture selection on nonsynonymous mutations.

#### Traditional YNGKP (or Goldman-Yang) models

To enable comparison of an ExpCM with non-site-specific substitution models, phydms implements several of these more traditional models. Here these models are referred to as YNGKP as they are variants of the Goldman-Yang style models described by Yang et al. (2000). Note that sometimes in the literature these models are called GY94 rather than YNGKP; however here we use the name YNGKP to explicitly emphasize that we are using the model variants delineated by Yang et al. (2000) rather than the original variants described in Goldman & Yang (1994). The M0 and M5 YNGKP models are implemented in phydms. The M0 variant optimizes a single dN/dS ratio (*ω*) and so is comparable with the basic ExpCM, while the M5 variant draws *ω* from a gamma distribution and so is comparable to an ExpCM with the –gammaomega option. The equilibrium codon frequencies are calculated empirically after correcting for stop codons as described by Pond et al. (2010) (the CF3X4 method). The M0 variant has 11 parameters (nine empirical nucleotide frequencies plus *ω* and *κ*), while the M5 variant has 12 parameters (*ω* is replaced by the two gamma-distribution parameters).

YNGKP models are less computationally expensive than an ExpCM since they are not site-specific. Therefore, YNGKP models are faster than the ExpCM in phydms. However, phydms is *not* optimized for maximal speed with YNGKP models, so if you are only using those models then consider using PAML (Yang, 2007) or HyPhy (Pond, Frost & Muse, 2005).

### Gradient-based optimization of the likelihood

Given one of the substitution models described above and a fixed phylogenetic tree topology, phydms numerically optimizes the model parameters and branch lengths to their maximum likelihood values via the Felsenstein pruning algorithm (Felsenstein, 1981). Numerical optimization generally requires fewer steps if the gradient of the objective function with respect to free parameters is computed explicitly (Gill, Murray & Wright, 1982), although this advantage can be offset by the cost of computing the gradient. We were unable to find clear published comparisons of the efficiency of phylogenetic optimization with and without an explicit gradient, although Kenney & Gu (2012) describe how the gradient (and Hessian matrix of second derivatives) can be computed.

We chose to use gradient-based optimization for phydms under the supposition that it might be more efficient. The first derivatives with respect to branch lengths and virtually all the model parameters can be computed analytically, propagated through the matrix exponentials using the formula provided by Kalbeisch & Lawless (1985), and evaluated along the tree by applying the chain rule to the Felsenstein pruning algorithm. For the ExpCM empirical nucleotide frequencies *ϕ*_*w*_ and the gamma-distributed *ω*, we used the numerical finite-difference method to compute small portions of the derivatives for which we could not derive analytic results. File S1 details how phydms computes the likelihood and its gradient.

For the optimization, we used the limited-memory BFGS optimizer with bounds (Byrd et al., 1995; Zhu et al., 1997; Morales & Nocedal, 2011). This optimizer uses the gradient, although this can be turned off with the –nograd option to phydms (doing so is *not* recommended as the accuracy of phydms without gradients has not been extensively tested, and the limited-memory BFGS optimizer may not perform well without gradients). Rather than optimizing model parameters and branch lengths simultaneously, phydms takes an iterative approach. First the model parameters are simultaneously optimized along with a single scaling parameter that multiplies all branch lengths. After this optimization has converged, all branch lengths are simultaneously optimized while holding the model parameters constant. This process is repeated until further optimization leads to negligible improvement in the likelihood. Note that simultaneous optimization of all branch lengths appears to be the minority approach in phylogenetics software (Bryant, Galtier & Poursat, 2005) and is said by Yang (2000) to be less efficient than one-at-a-time optimization; however, we found it to work effectively on the trees that we tested. The rationale for iterating between model parameters and branch lengths is that optimization of the former is more costly in terms of the gradient computation. If you simply want to scale branch lengths by a single parameter rather than optimize them, you can use the –brlen scale option. In other contexts, scaling but not individually optimizing branch lengths has been shown to reduce runtime with little effect on final model parameters if the initial tree is reasonably accurate (Yang, 2000; Pond & Frost, 2005).

### Design and implementation of phydms

The phydms software is written in Python. Most of the numerical computation is performed with numpy and scipy, and a few parts of the code are written in compiled C extensions created via cython. The limited-memory BFGS optimizer used by phydms is the one provided with scipy.optimize. The most computationally costly part of the optimization performed by phydms is the matrix-matrix multiplication performed when computing exponentials of the transition matrix, and the second most costly part is the matrix–vector multiplication performed while implementing the Felsenstein pruning algorithm. Both these steps are performed using BLAS subroutines called via scipy.

In addition to the core phydms program, the software is distributed with auxillary programs that make it easy to prepare alignments (phydms_prepalignment) and run multiple models for comparison (phydms_comprehensive). Importantly, phydms currently does *not* infer phylogenetic tree topologies, but rather optimizes branch lengths and model parameters given a topology. The tree topology must therefore be inferred using another program such as RAxML (Stamatakis, 2014) with a simpler substitution model.

### Visualization of the results with logoplots

It is often instructive to visualize the amino-acid preferences that are used to inform an ExpCM, as these preferences determine the unique properties of the models. In addition, visualization can help understand how the stringency parameter *β* optimized by phydms re-scales the preferences to increase concordance with natural selection. To aid such visualizations, phydms comes with an auxiliary program (phydms_logoplot) that renders the amino-acid preferences in the form of logoplots via the weblogo libraries (Crooks et al., 2004). The ‘RESULTS’ section shows example logoplots.

### Computer code

The phydms software is freely available on GitHub at https://github.com/jbloomlab/phydms. Detailed documentation is at http://jbloomlab.github.io/phydms. Analyses in this paper used versions of phydms ranging from 2.0.0 to 2.0.5.

## Results

### Testing phydms on two different genes

In the next few subsections, we describe example applications of phydms to real-world data sets. Specifically, we use phydms to compare deep mutational scanning measurements to natural sequence evolution for two genes: influenza hemagglutinin (HA) and *β*-lactamase. We choose these genes because there are multiple published deep mutational scanning datasets for each.

Analysis with an ExpCM requires three pieces of input data: the experimentally measured amino-acid preferences, an alignment of naturally occurring gene sequences, and a phylogenetic tree topology. The tree topology can be inferred from the sequence alignment. But like most other software for codon-based phylogenetic analyses (Pond, Frost & Muse, 2005; Yang, 2007), phydms is not designed to infer the tree topology. Instead, it provides easy ways to infer the tree topology using RAxML (Stamatakis, 2014).

**Figure 2:**
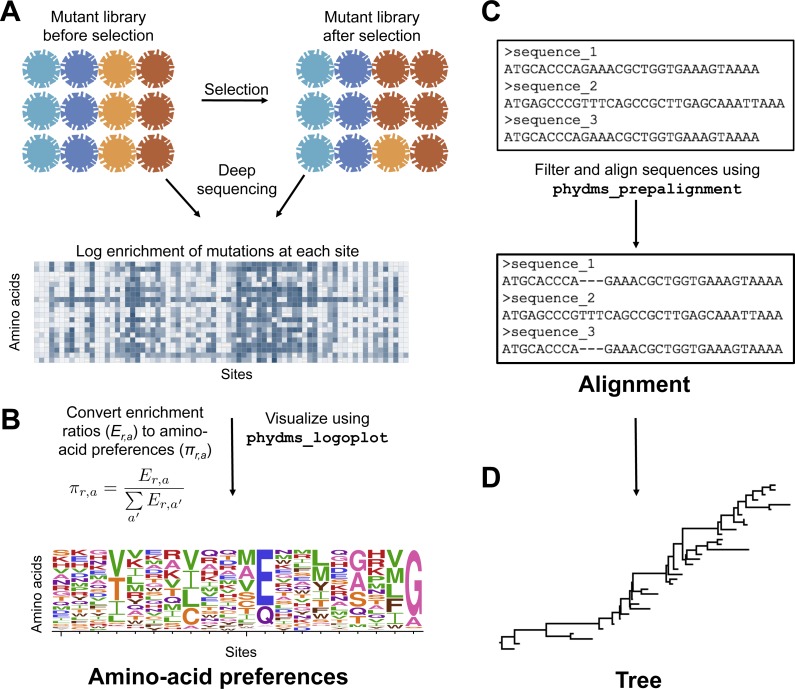
Workflow for preparing input data to phydms. Analysis with phydms requires amino-acid preferences measured by deep mutational scanning, a codon-level alignment of naturally occurring sequences, and a phylogenetic tree topology. (A) Deep mutational scanning involves performing a functional selection on a library of mutant genes, and using deep sequencing to quantify the enrichment or depletion of each mutation (relative to wildtype) after selection. (B) The amino-acid preferences used by the ExpCM can be calculated by normalizing the enrichment ratios for mutations to sum to one at each site. (C) We created a filtered, codon-level alignment of naturally occurring sequences using phydms_prepalignment. (D) We used phydms_comprehensive to automatically generate a tree topology from the filtered alignment using RAxML.

To prepare the required input data, we followed the workflow in Fig. 2. The deep mutational scanning experiments on HA (Thyagarajan & Bloom, 2014; Doud & Bloom, 2016) directly reported amino-acid preferences. However, the two deep mutational scanning experiments on *β*-lactamase (Firnberg et al., 2014; Stiffler, Hekstra1 & Ranganathan, 2015) reported enrichment ratios for each mutation rather than amino-acid preferences. There is a simple relationship between enrichment ratios and amino-acid preferences: the preferences are the enrichment ratios after normalizing the values to sum to one at each site, enabling easy conversion between the two data representations (Fig. 2).

We also created codon-level alignments of naturally occurring HA and *β*-lactamase sequences using phydms_prepalignment. The alignments were trimmed to contain only sites for which amino-acid preferences were experimentally measured. Table 1 summarizes basic information about these alignments.

**Table 1:** Alignments and deep mutational scanning (DMS) studies for HA and *β*-lactamase.

Gene	DMS studies	Residues in protein	Residues with DMS data	Sequences in alignment
HA	Doud & Bloom (2016), Thyagarajan & Bloom (2014)	565	564	34
*β*-lactamase	Stiffler, Hekstra1 & Ranganathan (2015), Firnberg et al. (2014)	285	263	50

**Table 2:** Fitting of an ExpCM informed by the HA preferences from Doud & Bloom (2016) to natural sequences using phydms_comprehensive. Full code, data, and results are in File S2.

Model	ΔAIC	Log likelihood	Number of parameters	Parameter values
ExpCM	0.0	−4877.7	6	*β* = 2.11, *κ* = 5.14, *ω* = 0.52
ExpCM, averaged preferences	2090.6	−5922.9	6	*β* = 0.68, *κ* = 5.36, *ω* = 0.22
YNGKP_M5	2113.5	−5928.4	12	*α*_*ω*_ = 0.30, *β*_*ω*_ = 1.42, *κ* = 4.68
YNGKP_M0	2219.6	−5982.5	11	*κ* = 4.61, *ω* = 0.20

### Test if deep mutational scanning is informative about natural selection

A first simple test is whether the deep mutational scanning experiment provides any information that is relevant to natural selection on the gene in question. This can be determined by testing whether an ExpCM that uses the experimental data outperforms a substitution model that is agnostic to the site-specific preferences measured in the experiments.

To perform such a test, we used phydms_comprehensive to fit several substitution models to the alignment of HA sequences. This program automatically generates a phylogenetic tree topology from the alignment using RAxML (Stamatakis, 2014). It then fits an ExpCM (in this case informed by the deep mutational scanning data in Doud & Bloom (2016)) as well as several substitution models that do not utilize site-specific experimental information. The analysis was performed by running the following command on the input data in File S2:

phydms_comprehensive results/ HA_alignment.fasta HA_Doud_prefs.csv

Table 2 lists the four tested substitution models: the ExpCM, an ExpCM with the amino-acid preferences averaged across sites, and the M0 and M5 variants of the standard Goldman-Yang style substitution models (Yang et al., 2000). (Because these variants were originally described by Yang, Nielsen, Goldman, and Krabbe-Pedersen, they are referred to here as YNGKP models; note that other literature sometimes uses the alternative acronym GY94.) The ExpCM with averaged preferences is a sensible control because the averaging eliminates any experimental information specific to individual sites in the protein. Because the models have different numbers of free parameters, they are best compared using Akaike Information Criterion (AIC) (Posada & Buckley, 2004), which compares log likelihoods after correcting for the number of free parameters. Table 2 shows that the ExpCM has a much smaller AIC than the other models (ΔAIC >2000 for all other models). Therefore, the experimentally measured amino-acid preferences contain information about natural selection on HA, since a substitution model informed by these preferences greatly outperforms models that do not utilize the experimental information.

### Re-scale deep mutational scanning data to stringency of natural selection

Even if a deep mutational scanning experiment measures the authentic natural selection on a gene, the stringency of selection in the experiment is not expected to match the stringency of selection in nature. Differences in the stringency of selection can be captured by the ExpCM stringency parameter *β*. If selection in nature prefers the same amino acids as the selection in lab but with greater stringency, *β* will be fit to a value >1. Conversely, if selection in nature does not prefer the lab-favored mutations with as much stringency as the deep mutational scan, *β* will be fit to a value <1. Table 2 shows that an ExpCM for HA informed by the experiments in (Doud & Bloom, 2016) has *β* = 2.11, indicating that natural selection favors the experimentally preferred amino acids with higher stringency than selection in the lab.

**Figure 3:**
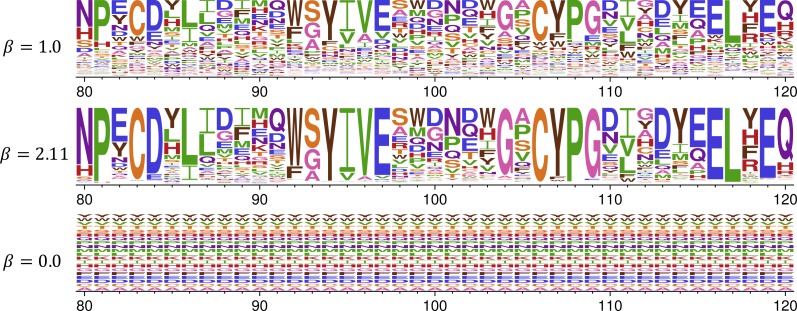
Re-scaling of amino-acid preferences to reflect the stringency of selection in nature. Analysis with phydms optimizes a stringency parameter *β* that relates the stringency of selection for preferred amino acids in the deep mutational scanning experiment to that in nature. When *β* = 1, the favored amino-acids are preferred in nature with the same stringency as during the experimental selections in the lab. When *β* > 1, selection in nature prefers the same amino acids as selection in lab but with greater stringency. When *β* < 1, selection in nature has less preference than the experiments for mutations favored in the lab, and when *β* = 0 then all site-specific information is lost. The actual optimized stringency parameter for HA reported in Table 2 is *β* = 2.11. We generated the logoplots shown above from the input data in File S3 with the following commands: phydms_logoplot HA_Doud_1.pdf –prefs HA_Doud_prefs_short.csv phydms_logoplot HA_Doud_2_11.pdf –prefs HA_Doud_prefs_short.csv –stringency 2.11 phydms_logoplot HA_Doud_0.pdf –prefs HA_Doud_prefs_short.csv –stringency 0.

The effect of this stringency re-scaling of the preferences can be visualized using phydms_logoplot as shown in Fig. 3. Re-scaling by the optimal stringency parameter of 2.11 exaggerates the selection for experimentally preferred amino acids. Conversely, if the analysis had fit a stringency parameter <1, this would have flattened the experimental measurements, and when *β* = 0 all information from the experiments is lost (Fig. 3). Because selection in the lab can probably never be tuned to exactly match that in nature, stringency re-scaling is a valuable method to standardize measurements across experiments.

### Compare how well different experiments capture natural selection

The amino-acid preferences for HA and *β*-lactamase have each been measured by two independent experiments. For each gene, which of these experiments better captures natural selection?

We can address this question by comparing ExpCM’s informed by each experiment. For *β*-lactamase, this means comparing the preferences measured by Stiffler, Hekstra1 & Ranganathan (2015) to those measured by Firnberg et al. (2014). We did this with phydms_comprehensive by running the following command on the input data in File S4:

phydms_comprehensive results/ betaLactamase_alignment.fasta betaLactamase_Stiffler_prefs.txt betaLactamase_Firnberg_prefs.txt

Table 3 shows that an ExpCM informed by the data of Stiffler, Hekstra1 & Ranganathan (2015) outperform an ExpCM informed by the data of Firnberg et al. (2014), with a ΔAIC of 96.2. Therefore, the former experiment better reflects natural selection on *β*-lactamase. However, both experiments are informative, as both greatly outperform traditional YNGKP models.

**Table 3:** Comparison of multiple *β*-lactamase deep mutational scanning results using phydms_comprehensive. Full code, data, and results are in File S4.

Model	ΔAIC	Log likelihood	Number of parameters	Parameter values
ExpCM, Stiffler preferences	0.0	−2581.3	6	*β* = 1.31, *κ* = 2.67, *ω* = 0.72
ExpCM, Firnberg preferences	96.2	−2629.4	6	*β* = 2.42, *κ* = 2.60, *ω* = 0.63
YNGKP_M5	739.2	−2944.9	12	*α*_*ω*_ = 0.30, *β*_*ω*_ = 0.49, *κ* = 3.02
YNGKP_M0	841.0	−2996.8	11	*κ* = 2.39, *ω* = 0.28

We made a similar comparison of the two deep mutational scans of HA. As summarized in Table 4 (and detailed in File S5), the deep mutational scanning of Doud & Bloom (2016) better describes the natural evolution than the experiments of Thyagarajan & Bloom (2014) (ΔAIC of 44.2). Again, both experiments are clearly informative, as both greatly outperform the YNGKP models.

**Table 4:** Comparison of multiple HA deep mutational scanning results using phydms_comprehensive. Full code, data, and results are in File S5.

Model	ΔAIC	Log likelihood	Number of parameters	Parameter values
ExpCM, Doud preferences	0.0	−4877.7	6	*β* = 2.11, *κ* = 5.14, *ω* = 0.52
ExpCM, Thyagarajan preferences	44.2	−4899.7	6	*β* = 1.72, *κ* = 4.94, *ω* = 0.55
YNGKP_M5	2113.5	−5928.4	12	*α*_*ω*_ = 0.30, *β*_*ω*_ = 1.42, *κ* = 4.68
YNGKP_M0	2219.6	−5982.5	11	*κ* = 4.61, *ω* = 0.20

**Figure 4:**
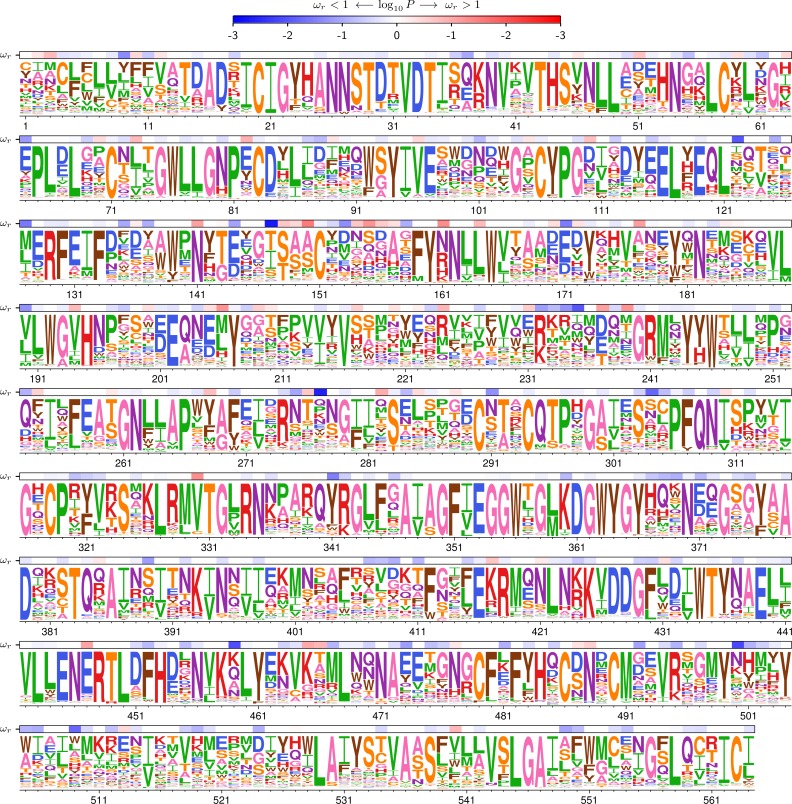
Identifying sites of diversifying selection. The phydms option –omegabysite fits a site-specific value for *ω*_*r*_, which gives the relative rate of non-synonymous to synonymous substitutions at site *r* after accounting for the selection due to the amino-acid preferences. This figure shows the results of such an analysis for HA. The overlay bar represents the strength of evidence for *ω*_*r*_ being greater (red) or less (blue) than one. Because this approach accounts for the constraints due to the amino-acid preferences, it can identify sites evolving faster than expected even if their absolute relative rates of nonysnonymous to synonymous substitutions do not significantly differ from one. The logoplot in this figure uses the stringency parameter value of *β* = 2.11, and was generated by running the following command on the data in File S3: phydms_logoplot results/omegabysite.pdf –prefs HA_Doud_prefs.csv –omegabysite results/omegabysite.txt –stringency 2.11 –minP 0.001. In this figure, the HA sequence is numbered sequentially beginning with 1 for the first site with deep mutational scanning data, which is the second residue in the protein.

### Identify sites of diversifying selection

In some cases, a few sites may evolve differently in nature than expected from the experiments in the lab. For instance, sites under diversifying selection for amino-acid change will experience more nonsynonymous substitutions than expected given the experimentally measured amino-acid preferences. Such sites can be identified by using the –omegabysite option to fit a parameter *ω*_*r*_ that gives the relative rate of nonsynonymous to synonymous substitutions after accounting for the experimentally measured preferences for each site *r* (Bloom, 2017). If the preferences capture all the selection on amino acids, then we expect *ω*_*r*_ = 1. Sites with *ω*_*r*_ > 1 are under diversifying selection for amino-acid change, while sites with *ω*_*r*_ < 1 are under additional purifying selection not measured in the lab.

We tested for diversifying selection in HA by running the following command on the data in File S6:

phydms HA_alignment.fasta HA_RAxML_tree.newick ExpCM_HA_Doud_prefs.csv results/ –omegabysite

The results are visualized in Fig. 4. While most sites are evolving with *ω*_*r*_ not significantly different from one, some sites show evidence of *ω*_*r*_ > 1. As described in Bloom (2017), these sites may be under diversifying selection due to immune pressure. Overall, this analysis shows how phydms can identify sites evolving differently in nature than expected from experiments in the lab.

### phydms has superior computational performance to existing alternatives

Our rationale for developing phydms was to enable the analyses described above to be performed more easily than with existing software. To validate the improved computational performance, we compared phydms (version 2.0.0) to alternative programs that have been used to fit an ExpCM. The comparisons used the HA sequences described in Table 1 with an ExpCM informed by the deep mutational scanning in Doud & Bloom (2016), and were performed on a single core of a 2.6 GHz Intel Xeon CPU.

Table 5 shows the results. With default settings, phydms took 10 min to optimize the model parameters and branch lengths. This runtime could be decreased by scaling the branch lengths by a single parameter rather than optimizing them individually (–brlen scale option); other work has shown that when the initial tree is reasonably accurate, this approximation can improve runtime while only slightly affecting model fit (Yang, 2000; Pond & Frost, 2005). Fitting the nucleotide frequency parameters *ϕ*_*w*_ (–fitphi option) rather than determining them empirically doubled the runtime. The log likelihood and values of the model parameters *β* and *ω* were nearly identical for all three of these settings. The gradient-based optimization is important: using phydms without gradients (–nograd option) increased the runtime over 5-fold while also yielding a poorer log likelihood.

Two alternative programs have previously been used to fit an ExpCM. Bloom (2014a) and Bloom (2014b) used a Python program (phyloExpCM) to run HyPhy to optimize an ExpCM similar to the ones used here. Bloom (2017) used an old version of phydms to fit an ExpCM identical to the ones here using the Bio++ libraries (Guéguen et al., 2013). We ran both these programs on the HA data set, using phyloExpCM version 0.3 with HyPhy version 2.22, and phydms version 1.3.0 with Bio++. Table 5 shows that these programs were ∼100-fold and ∼200-fold slower than phydms with default settings. A small portion of the slower runtime is because these earlier implementations cannot calculate empirical nucleotide frequency *ϕ*_*w*_ parameters; however they remain much slower than phydms even when these parameters are fit. Note that Table 5 may overestimate the computational advantage of phydms over HyPhy in some situations, since HyPhy code but not phydms can in principle be written to enable the use of multiple cores. Divining the reasons for the performance differences was not possible, as the programs differ completely in their implementations. But reassuringly, all programs yielded similar model parameters *β* and *ω* despite independent implementations of the likelihood calculations and the optimization.

**Table 5:** Comparison of phydms to alternative software for optimizing a tree of 34 HA sequences HyPhy and Bio++ use models that fit *ϕ*, whereas by default phydms determines *ϕ*_*w*_ empirically. Log likelihoods are not expected to be identical across software. Full code, data, and results are in File S7.

Software	Runtime (min)	Log likelihood	*β*	*ω*
phydms, scale branches	7.8	−4877.9	2.11	0.52
phydms, default settings	10.5	−4877.7	2.11	0.52
phydms, fit *ϕ* values	23.2	−4876.5	2.11	0.53
phydms, no gradient	52.8	−4894.0	2.13	0.57
Bio++ via old phydms	962.6	−4880.6	2.09	0.53
HyPhy via phyloExpCM	2102.0	−4908.4	2.11	0.57

The analyses above used relatively small alignments of 34 or 50 sequences (Table 1). To test how the performance of phydms changed with alignment size, we analyzed HA alignments ranging from 34 to 108 sequences. As shown in Table 6, the runtime increased with alignment size, but remained under an hour even for the largest alignment. The inferred model parameter values also remained relatively constant as the size of the HA alignment increased (Table 6).

**Table 6:** Comparison of parameter values and runtimes for HA alignments of different sizes using default phydms settings. The alignments are different than those used for the other HA analyses in this paper thus explaining the slightly different parameter values. The alignments, full code, data, and results are in File S8.

Sequences in alignment	Runtime (min)	*β*	*ω*
34	14.5	1.97	0.42
62	37.2	1.92	0.45
85	41.0	1.87	0.48
104	51.2	1.87	0.49

## Discussion

We have described a new software package that facilitates efficient analyses with phylogenetic substitution models informed by deep mutational scanning experiments. This software, phydms, can quantitatively compare deep mutational scanning measurements to selection on genes in nature. It can re-scale deep mutational scanning data to account for differences in the stringency of selection between the lab and nature, identify sites evolving differently in nature than expected from the experiments, and compare how well different experiments on the same gene describe natural selection.

The ability to perform these comparisons is useful because the rationale for many deep mutational scanning experiments is to provide information about the effects of mutations on genes in nature. For instance, there are many ways to design an experiment, and it is often not obvious which choice is best if the goal is to make the experiment reflect natural selection. Using phydms, it is possible to quantitatively compare how well different experiments describe natural selection. Likewise, it is often useful to know if specific sites in a gene are evolving differently in nature than expected from experiments in the lab. Algorithms implemented in phydms makes statistically rigorous identification of these sites possible.

The speed and ease of use of phydms makes these analyses practical for real datasets. As deep mutational scanning data become available for an increasing number of genes, phydms will facilitate comparison of the experimental measurements to selection in nature.

##  Supplemental Information

10.7717/peerj.3657/supp-1 File S1 This PDF contains details of the calculations of the likelihood and its gradient as implemented in *phydms*

10.7717/peerj.3657/supp-2 File S2 This ZIP file contains the code, input data, and full results of the *phydms* analysis summarized in Table 2

10.7717/peerj.3657/supp-3 File S3 This ZIP file contains the code, input data, and full results of the stringency parameter comparison with *phydms_logoplot* summarized in Figure 3

10.7717/peerj.3657/supp-4 File S4 This ZIP file contains the code, input data, and full results of the multiple beta-lactamase deep mutational scan comparison summarized in Table 3

10.7717/peerj.3657/supp-5 File S5 This ZIP file contains the code, input data, and full results of the multiple HA deep mutational scan comparison summarized in Table 4

10.7717/peerj.3657/supp-6 File S6 This ZIP file contains the code, input data, and full results of the *phydms –omegabysite* analysis summarized in Figure 4

10.7717/peerj.3657/supp-7 File S7 This ZIP file contains the code, input data, and full results of the program runtime comparison summarized in Table 5

10.7717/peerj.3657/supp-8 File S8 This ZIP file contains the code, input data, and full results of the alignment size comparison summarized in Table 6

## Additional Information and Declarations

### Data Availability

The following information was supplied regarding data availability: All data and code are available on GitHub: https://github.com/jbloomlab/phydms. Detailed documentation is at http://jbloomlab.github.io/phydms. Hilton, Doud, and Bloom, bioRxiv, 2017.
http://www.biorxiv.org/content/early/2017/03/29/121830.
