# Supplementary material for: phydms: software for phylogenetic analyses informed by deep mutational scanning
Source: PeerJ. 2017 Jul 31;5:e3657. doi: 10.7717/peerj.3657 (PMC5541924; doi:10.7717/peerj.3657)
Supplement: File S1 [file peerj-05-3657-s001.pdf]

Likelihood and gradient

Jesse D. Bloom and Sarah K. Hilton

March 20, 2017

This document contains some information on the numerical implementation of `phydms` (version 2.0.0), particularly in regards to how the likelihoods and its derivatives are computed. It may be of help in trying to understand the code.

Contents

1 *ExpCM* substitution model parameters and derivatives **2**

2 *ExpCM* stationary state and derivatives **3**

3 *ExpCM* with empirical nucleotide frequencies **5**

4 *ExpCM* with empirical nucleotide frequencies and diversifying pressure **7**

5 *ExpCM* with the preferences as free parameters **8**
- 5.1 `ExpCM_fitprefs` implementation 9
- 5.2 `ExpCM_prefs2` implementation 10

6 Regularizing preferences for *ExpCM* with preferences as free parameters **11**
- 6.1 Inverse-quadratic prior 11
  - 6.1.1 `ExpCM_fitprefs` implementation 12
  - 6.1.2 `ExpCM_fitprefs2` implementation 12

7 *YNGKP_M0* model **12**

8 Exponentials of the substitution matrix and derivatives **13**

9 Scaling the branch lengths with a mutation rate **14**

10 Calculating the likelihood and derivatives on a tree **15**

11 Scaling to avoid numerical underflow **17**

12 Units of tree branch lengths **18**

13 Models with gamma-distributed ω **18**

14 Derivatives with respect to branch lengths **19**

15 Optimization **20**

1 *ExpCM* substitution model parameters and derivatives

We begin by considering the basic *ExpCM* substitution model described in [1]. $P_{r,xy}$ gives the substitution rate from codon x to codon $y \neq x$ at site r, and is defined by

$$P_{r,xy} = \begin{cases} Q_{xy} \times F_{r,xy} & \text{if } x \neq y, \\ -\sum\limits_{z \neq x} F_{r,xz} Q_{xz} & \text{if } x = y. \end{cases} \tag{1}$$

where Q_{xy} is the rate of mutation from codon x to y and is defined by

$$Q_{xy} = \begin{cases} \phi_w & \text{if } x \text{ is converted to } y \text{ by a single-nucleotide transversion to } w, \\ \kappa\phi_w & \text{if } x \text{ is converted to } y \text{ by a single-nucleotide transition to } w, \\ 0 & \text{if } x \text{ and } y \text{ differ by more than one nucleotide,} \end{cases} \tag{2}$$

where κ is the transition-transversion ratio and ϕ_w is the expected frequency of nucleotide w in the absence of selection on amino-acid mutation (and so is subject to the constraint $1 = \sum_w \phi_w$).

The "fixation probability" $F_{r,xy}$ of the mutation from x to y is

$$F_{r,xy} = \begin{cases} 1 & \text{if } \mathcal{A}(x) = \mathcal{A}(y) \\ \omega & \text{if } \mathcal{A}(x) \neq \mathcal{A}(y) \text{ and } \pi_{r,\mathcal{A}(x)} = \pi_{r,\mathcal{A}(y)} \\ \omega \times \frac{-\beta \ln\left(\pi_{r,\mathcal{A}(x)} / \pi_{r,\mathcal{A}(y)}\right)}{1 - \left(\pi_{r,\mathcal{A}(x)} / \pi_{r,\mathcal{A}(y)}\right)^{\beta}} & \text{otherwise.} \end{cases} \tag{3}$$

where $\pi_{r,a}$ is the preference of site r for amino acid a, $\mathrm{A}(x)$ is the amino acid encoded by codon x, β is the *stringency parameter*, and ω is a relative rate of nonsynonymous to synonymous mutations after accounting for the selection encapsulated by the preferences.

We define a variable transformation of the four nucleotide frequency parameters ϕ_w (three of which are unique). This transformation aids in numerical optimization. Specifically, we number the four nucleotides in alphabetical order so that $w = 0$ denotes A, $w = 1$ denotes C, $w = 2$ denotes G, and $w = 3$ denotes T. We then define the three free variables η_0, η_1, and η_2, all of which are constrained to fall between zero and one. For notational convenience in the formulas below, we also define $\eta_3 = 0$; note however that η_3 is not a free parameter, as it is always zero. We define ϕ_w in terms of these η_i variables by

$$\phi_w = \left(\prod_{i=0}^{w-1} \eta_i\right) (1 - \eta_w) \tag{4}$$

or conversely

$$\eta_w = 1 - \phi_w / \left(\prod_{i=0}^{w-1} \eta_i\right). \tag{5}$$

Note that setting $\eta_w = \frac{3-w}{4-w}$ makes all of the ϕ_w values equal to $\frac{1}{4}$.

The derivatives are:

$$\frac{\partial \phi_w}{\partial \eta_i} = \begin{cases} \left(\prod_{j=0}^{i-1} \eta_j\right) \left(\prod_{j=i+1}^{w-1} \eta_j\right) (1 - \eta_w) = \frac{\phi_w}{\eta_i} & \text{if } i < w \\ -\prod_{j=0}^{w-1} \eta_j = \frac{\phi_w}{\eta_i - 1} & \text{if } i = w \\ 0 & \text{if } i > w \end{cases} \tag{6}$$

$$= \begin{cases} \frac{\phi_w}{\eta_i - \delta_{iw}} & \text{if } i \leq w, \\ 0 & \text{otherwise,} \end{cases} \tag{7}$$

where δ_{ij} is the Kronecker-delta, equal to 1 if $i = j$ and 0 otherwise. Given these definitions, the free parameters in and *ExpCM* model are κ, η_0, η_1, η_2, β, and ω.

Here are the derivatives of $P_{r,xy}$ with respect to each of these parameters:

$$\frac{\partial P_{r,xy}}{\partial \kappa} = \begin{cases} \frac{P_{r,xy}}{\kappa} & \text{if } x \text{ is converted to } y \text{ by a transition of a nucleotide to } w, \\ 0 & \text{if } x \text{ and } y \text{ differ by something other than a single transition,} \\ -\sum\limits_{z \neq x} \frac{\partial P_{r,xz}}{\partial \kappa} & \text{if } x = y. \end{cases} \tag{8}$$

$$\frac{\partial P_{r,xy}}{\partial \eta_i} = \begin{cases} \frac{P_{r,xy}}{\phi_w} \frac{\partial \phi_w}{\partial \eta_i} = \frac{P_{r,xy}}{\eta_i - \delta_{iw}} & \text{if } x \text{ is converted to } y \text{ by a single-nucleotide mutation to } w \geq i, \\ 0 & \text{if } i > w \text{ or } x \text{ and } y \text{ differ by more than one nucleotide,} \\ -\sum\limits_{z \neq x} \frac{\partial P_{r,xz}}{\partial \eta_i} & \text{if } x = y. \end{cases} \tag{9}$$

$$\frac{\partial P_{r,xy}}{\partial \omega} = \begin{cases} 0 & \text{if } \mathrm{A}(x) = \mathrm{A}(y) \text{ and } x \neq y \\ \frac{P_{r,xy}}{\omega} & \text{if } \mathrm{A}(x) \neq \mathrm{A}(y), \\ -\sum\limits_{z \neq x} \frac{\partial P_{r,xz}}{\partial \omega} & \text{if } x = y. \end{cases} \tag{10}$$

$$\frac{\partial P_{r,xy}}{\partial \beta} = \begin{cases} 0 & \text{if } \mathrm{A}(x) = \mathrm{A}(y) \text{ and } x \neq y, \\ 0 & \text{if } \pi_{r,\mathrm{A}(x)} = \pi_{r,\mathrm{A}(y)} \text{ and } x \neq y, \\ \frac{P_{r,xy}}{\beta} + P_{r,xy} \frac{\left(\pi_{r,\mathrm{A}(x)}/\pi_{r,\mathrm{A}(y)}\right)^{\beta} \times \ln\left(\pi_{r,\mathrm{A}(x)}/\pi_{r,\mathrm{A}(y)}\right)}{1 - \left(\pi_{r,\mathrm{A}(x)}/\pi_{r,\mathrm{A}(y)}\right)^{\beta}} & \text{if } \mathrm{A}(x) \neq \mathrm{A}(y), \\ -\sum\limits_{z \neq x} \frac{\partial P_{r,xz}}{\partial \beta} & \text{if } x = y. \end{cases} \tag{11}$$

2 *ExpCM* stationary state and derivatives

The stationary state of the substitution model defined by $P_{r,xy}$ is

$$p_{r,x} = \frac{q_x f_{r,x}}{\sum_z q_z f_{r,z}} \tag{12}$$

where

$$f_{r,x} = \left(\pi_{r,\mathrm{A}(x)}\right)^{\beta} \tag{13}$$

and

$$q_x = \phi_{x_0}\phi_{x_1}\phi_{x_2} \tag{14}$$

where x_0, x_1, and x_2 are the nucleotides at the first, second, and third positions of codon x.

The derivatives of the stationary state with respect to κ and ω are zero as these do not affect that state, so:

$$\frac{\partial p_{r,x}}{\partial \kappa} = \frac{\partial p_{r,x}}{\partial \omega} = 0. \tag{15}$$

The stationary state is sensitive to the value of β, with derivative:

$$\begin{aligned}
\frac{\partial p_{r,x}}{\partial \beta} &= \frac{p_{r,x}\left[\ln\left(\pi_{r,\mathrm{A}(x)}\right)\left(\sum_z f_{r,z}q_z\right) - \sum_z \ln\left(\pi_{r,\mathrm{A}(z)}\right) f_{r,z}q_z\right]}{\sum_z q_z f_{r,z}} && (16)\\
&= p_{r,x}\left(\ln\left(\pi_{r,\mathrm{A}(x)}\right) - \frac{\sum_z \ln\left(\pi_{r,\mathrm{A}(z)}\right) f_{r,z}q_z}{\sum_z q_z f_{r,z}}\right) && (17)\\
&= p_{r,x}\left(\ln\left(\pi_{r,\mathrm{A}(x)}\right) - \sum_z \ln\left(\pi_{r,\mathrm{A}(z)}\right) p_{r,z}\right) && (18)
\end{aligned}$$

The stationary state is also sensitive to the values of η_0, η_1, and η_2:

$$\begin{aligned}
\frac{\partial p_{r,x}}{\partial \eta_i} &= \frac{f_{r,x}\frac{\partial q_x}{\partial \eta_i}\left(\sum_z q_z f_{r,z}\right) - f_{r,x}q_x\left(\sum_z f_{r,z}\frac{\partial q_z}{\partial \eta_i}\right)}{\left(\sum_z q_z f_{r,z}\right)^2} && (19)\\
&= \frac{\partial q_x}{\partial \eta_i}\frac{p_{r,x}}{q_x} - p_{r,x}\frac{\sum_z f_{r,z}\frac{\partial q_z}{\partial \eta_i}}{\sum_z q_z f_{r,z}} && (20)
\end{aligned}$$

where the $\frac{\partial q_x}{\partial \eta_i}$ terms are:

$$\begin{aligned}
\frac{\partial q_x}{\partial \eta_i} &= \frac{\partial \phi_{x_0}}{\partial \eta_i}\phi_{x_1}\phi_{x_2} + \frac{\partial \phi_{x_1}}{\partial \eta_i}\phi_{x_0}\phi_{x_2} + \frac{\partial \phi_{x_2}}{\partial \eta_i}\phi_{x_0}\phi_{x_1} && (21)\\
&= \sum_{j=0}^{2}\frac{\partial \phi_{x_j}}{\partial \eta_i}\prod_{k\neq j}\phi_{x_k} && (22)\\
&= q_x\sum_{j=0}^{2}\frac{1}{\phi_{x_j}}\frac{\partial \phi_{x_j}}{\partial \eta_i} && (23)\\
&= q_x\sum_{j=0}^{2}\frac{\mathrm{bool}\left(i \leq x_j\right)}{\eta_i - \delta_{ix_j}} && (24)
\end{aligned}$$

where bool$(i \leq j)$ is 1 if $i \leq j$ and 0 otherwise, and so

$$\frac{\partial p_{r,x}}{\partial \eta_i} = p_{r,x} \left[\sum_{j=0}^{2} \frac{\text{bool}\left(i \leq x_j\right)}{\eta_i - \delta_{i x_j}} - \frac{\sum_z f_{r,z} q_z \sum_{j=0}^{2} \frac{\text{bool}(i \leq z_j)}{\eta_i - \delta_{i z_j}}}{\sum_z q_z f_{r,z}} \right] \tag{25}$$

$$= p_{r,x} \left[\sum_{j=0}^{2} \frac{\text{bool}\left(i \leq x_j\right)}{\eta_i - \delta_{i x_j}} - \frac{\sum_z p_{r,z} \sum_{j=0}^{2} \frac{\text{bool}(i \leq z_j)}{\eta_i - \delta_{i z_j}}}{\sum_z p_{r,z}} \right] \tag{26}$$

$$\tag{27}$$

3 *ExpCM* with empirical nucleotide frequencies

In the description above, the nucleotide frequencies ϕ_w are fit as three free parameters. Now let's consider the case where we instead calculate them empirically to give a stationary state that implies nucleotide frequencies that match those empirically observed in the alignment. This should be beneficial in terms of optimization because it reduces the number of model parameters that need to be optimized.

Let g_w be the empirical frequency of nucleotide w taken over all sites and sequences in the alignment. Obviously, $1 = \sum_w g_w$. We want to empirically set ϕ_w to some value $\hat{\phi}_w$ such that when $q_x = \hat{\phi}_{x_0} \hat{\phi}_{x_1} \hat{\phi}_{x_2}$ then

$$g_w = \frac{1}{L} \sum_r \sum_x \frac{1}{3} N_w\left(x\right) p_{r,x} \tag{28}$$

$$= \frac{1}{3L} \sum_r \frac{\sum_x N_w\left(x\right) f_{r,x} \prod_{k=0}^{2} \hat{\phi}_{x_k}}{\sum_y f_{r,y} \prod_{k=0}^{2} \hat{\phi}_{y_k}} \tag{29}$$

$$\tag{30}$$

where $N_w\left(x\right) = \sum_{k=0}^{2} \delta_{x_k,w}$ is the number of occurrence of nucleotide w in codon x, r ranges over all codon sites in the gene, x ranges over all codons, and k ranges over the first three nucleotides.

There are three independent g_w values and three independent $\hat{\phi}_w$ values (since $1 = \sum_w g_w \sum_w = \hat{\phi}_w$), so we have three equations and three unknowns. We could not solve the set of three equations analytically for the $\hat{\phi}_w$ values, so instead we use a non-linear equation solver to determine their values.

When using empirical nucleotide frequencies, we no longer need to calculate any derivatives with respect to η_i as we no longer have the η_i free parameters.

However, now the value of $\phi_w = \hat{\phi}_w$ depends on β via the $f_{r,x}$ parameters in Equation phihat. So we need new formulas for $\frac{\partial p_{r,x}}{\partial \beta}$ and $\frac{\partial P_{r,xy}}{\partial \beta}$ that accounts for this dependency.

Since we do not have an analytic expression for $\hat{\phi}_w$, we cannot compute $\frac{\partial \phi_w}{\partial \beta}$ analytically. But we can compute these derivatives numerically. This is done using a finite-difference method.

We now update the formula for $\frac{\partial P_{r,xy}}{\partial \beta}$ for the case when ϕ_w depends on β. In that case, we have:

$$\frac{\partial Q_{xy}}{\partial \beta} = \begin{cases} \frac{\partial \phi_w}{\partial \beta} & \text{if } x \text{ is converted to } y \text{ by a single-nucleotide transversion to } w, \\ \kappa \frac{\partial \phi_w}{\partial \beta} & \text{if } x \text{ is converted to } y \text{ by a single-nucleotide transition to } w, \\ 0 & \text{if } x \text{ and } y \text{ differ by more than one nucleotide,} \end{cases} \tag{31}$$

and

$$\frac{\partial F_{r,xy}}{\partial\beta} = \begin{cases} 0 & \text{if } \mathcal{A}(x)=\mathcal{A}(y) \\ 0 & \text{if } \mathcal{A}(x)\neq\mathcal{A}(y) \text{ and } \pi_{r,\mathcal{A}(x)}=\pi_{r,\mathcal{A}(y)} \\ \frac{F_{r,xy}\left(1-\frac{F_{r,xy}}{\omega}\left(\pi_{r,\mathrm{A}(x)}/\pi_{r,\mathrm{A}(y)}\right)^{\beta}\right)}{\beta} & \text{otherwise,} \end{cases} \tag{32}$$

so for all $x \neq y$, we have

$$\frac{\partial P_{r,xy}}{\partial\beta} = \frac{\partial\left(Q_{xy}\times F_{r,xy}\right)}{\partial\beta} \tag{33}$$

$$= Q_{xy}\frac{\partial F_{r,xy}}{\partial\beta} + F_{r,xy}\frac{\partial Q_{xy}}{\partial\beta} \tag{34}$$

$$= \left[\frac{\partial P_{r,xy}}{\partial\beta}\right]_{\text{free }\phi_w} + F_{r,xy}\frac{\partial Q_{xy}}{\partial\beta}. \tag{35}$$

where $\left[\frac{\partial P_{r,xy}}{\partial\beta}\right]_{\text{free }\phi_w}$ is the expression given by the equation for $\frac{\partial P_{r,xy}}{\partial\beta}$. When $x=y$, we have $\frac{\partial P_{r,xx}}{\partial\beta} = \sum\limits_{z\neq x} -\frac{\partial P_{r,xz}}{\partial\beta}$.

We also must update the formula in for $\frac{\partial P_{r,xy}}{\partial\beta}$ for the case where ϕ_w depends on β. We have:

$$\frac{\partial q_x}{\partial\beta} = \frac{\partial\left(\phi_{x_0}\phi_{x_1}\phi_{x_2}\right)}{\partial\beta} \tag{36}$$

$$= \sum_{j=0}^{2}\frac{\partial\phi_{x_j}}{\partial\beta}\prod_{k\neq j}\phi_{x_k} \tag{37}$$

$$= q_x\sum_{j=0}^{2}\frac{1}{\phi_{x_j}}\frac{\partial\phi_{x_j}}{\partial\beta} \tag{38}$$

and

$$\frac{\partial f_{r,x}}{\partial\beta} = f_{r,x}\left[\ln\left(\pi_{r,\mathrm{A}(x)}\right)\right]. \tag{39}$$

So:

$$\frac{\partial p_{r,x}}{\partial \beta} = \frac{\partial}{\partial \beta}\left(\frac{q_x f_{r,x}}{\sum_z q_z f_{r,z}}\right) \quad (40)$$

$$= \frac{\left(q_x \frac{\partial f_{r,x}}{\partial \beta} + f_{r,x}\frac{\partial q_x}{\partial \beta}\right)\sum_z q_z f_{r,z} - q_x f_{r,x}\sum_z\left(q_z \frac{\partial f_{r,z}}{\partial \beta} + f_{r,z}\frac{\partial q_z}{\partial \beta}\right)}{\left(\sum_z q_z f_{r,z}\right)^2} \quad (41)$$

$$= \frac{\left(q_x f_{r,x}\left[\ln\left(\pi_{r,\mathrm{A}(x)}\right)\right] + f_{r,x} q_x \sum\limits_{j=0}^{2}\frac{1}{\phi_{x_j}}\frac{\partial \phi_{x_j}}{\partial \beta}\right)\sum_z q_z f_{r,z} - q_x f_{r,x}\sum_z\left(q_z f_{r,z}\left[\ln\left(\pi_{r,\mathrm{A}(z)}\right)\right] + f_{r,z} q_z \sum\limits_{j=0}^{2}\frac{1}{\phi_{z_j}}\frac{\partial \phi_{z_j}}{\partial \beta}\right)}{\left(\sum_z q_z f_{r,z}\right)^2} \quad (42)$$

$$= p_{r,x}\frac{\left(\left[\ln\left(\pi_{r,\mathrm{A}(x)}\right)\right] + \sum\limits_{j=0}^{2}\frac{1}{\phi_{x_j}}\frac{\partial \phi_{x_j}}{\partial \beta}\right)\sum_z q_z f_{r,z} - \sum_z\left(q_z f_{r,z}\left[\ln\left(\pi_{r,\mathrm{A}(z)}\right)\right] + f_{r,z} q_z \sum\limits_{j=0}^{2}\frac{1}{\phi_{z_j}}\frac{\partial \phi_{z_j}}{\partial \beta}\right)}{\sum_z q_z f_{r,z}} \quad (43)$$

$$= p_{r,x}\left[\left[\ln\left(\pi_{r,\mathrm{A}(x)}\right)\right] + \sum_{j=0}^{2}\frac{1}{\phi_{x_j}}\frac{\partial \phi_{x_j}}{\partial \beta} - \sum_z p_{r,z}\left(\left[\ln\left(\pi_{r,\mathrm{A}(z)}\right)\right] + \sum_{j=0}^{2}\frac{1}{\phi_{z_j}}\frac{\partial \phi_{z_j}}{\partial \beta}\right)\right] \quad (44)$$

$$= \left[\frac{\partial p_{r,x}}{\partial \beta}\right]_{\text{free } \phi_w} + p_{r,x}\left[\sum_{j=0}^{2}\frac{1}{\phi_{x_j}}\frac{\partial \phi_{x_j}}{\partial \beta} - \sum_z p_{r,z}\sum_{j=0}^{2}\frac{1}{\phi_{z_j}}\frac{\partial \phi_{z_j}}{\partial \beta}\right] \quad (45)$$

where $\left[\frac{\partial p_{r,x}}{\partial \beta}\right]_{\text{free } \phi_w}$ is the expresssion given by the equation for $\frac{\partial p_{r,x}}{\partial \beta}$.

4 *ExpCM* with empirical nucleotide frequencies and diversifying pressure

The ω value in the previous models is the gene-wide relative rate of nonsynonymous to synonymous mutations after accounting for the differing preferences among sites. In some cases, it might be possible to specify *a priori* expections for the diversifying pressure at each site. For instance, viruses benefit from amino-acid change in sites targeted by the immune system and, consequently, these sites have a higher rate of amino-acid substitution than expected given their level of inherent functional constraint. We can incorporate our expectations for diversifying pressure at specific sites into the selection terms $F_{r,xy}$.

Let δ_r be the pre-determined diversifying pressure for amino-acid change at site r in the protein. A large positive value of δ_r corresponds to high pressure for amino-acid diversification, and negative value corresponds to expected pressure against amino-acid diversification beyond that captured in the amino-acid preferences. We then replace ω in Equation Frxy with the expression $\omega \times (1 + \omega_2 \times \delta_r)$, resulting in selection terms:

$$F_{r,xy} = \begin{cases} 1 & \text{if } \mathcal{A}(x) = \mathcal{A}(y) \\ \omega \times (1 + \omega_2 \times \delta_r) & \text{if } \mathcal{A}(x) \neq \mathcal{A}(y) \text{ and } \pi_{r,\mathcal{A}(x)} = \pi_{r,\mathcal{A}(y)} \\ \omega \times (1 + \omega_2 \times \delta_r) \times \frac{\ln\left(\left(\pi_{r,\mathcal{A}(y)}\right)^{\beta} / \left(\pi_{r,\mathcal{A}(x)}\right)^{\beta}\right)}{1 - \left(\left(\pi_{r,\mathcal{A}(x)}\right)^{\beta} / \left(\pi_{r,\mathcal{A}(y)}\right)^{\beta}\right)} & \text{otherwise.} \end{cases} \quad (46)$$

Whereas before ω reflected the elevation of non-synonymous substitution rate (averaged across the entire gene) beyond that expected given the amino-acid preferences, now ω reflects a gene-wide rate of elevated non-synonymous substitution after taking into account the expected sites of diversifying pressure (as represented by δ_r) weighted by $\omega_2 \times \delta_r$. These new selection terms in are identical the selection terms in the original equation for $F_{r,xy}$ when $\omega_2 = 0$.

To ensure a positive value of $\omega \times (1 + \omega_2 \times \delta_r)$, we constrain $\omega > 0$, $-1 < \omega_2 < \infty$, and $|\max_r \delta_r| \leq 1$.

We have added one more parameter, ω_2, so we need to add a new derivative, $\frac{\partial P_{r,xy}}{\partial \omega_2}$:

$$\frac{\partial P_{r,xy}}{\partial \omega_2} = \begin{cases} 0 & \text{if A}\left(x\right) = \text{A}\left(y\right) \text{ and } x \neq y \\ \omega \times \delta_r \times \frac{\ln\left(\left(\pi_{r,\mathcal{A}(y)}\right)^\beta / \left(\pi_{r,\mathcal{A}(x)}\right)^\beta\right)}{1-\left(\left(\pi_{r,\mathcal{A}(x)}\right)^\beta / \left(\pi_{r,\mathcal{A}(y)}\right)^\beta\right)} \times Q_{xy} & \text{if A}\left(x\right) \neq \text{A}\left(y\right), \\ -\sum\limits_{z \neq x} \frac{\partial P_{r,xy}}{\partial \omega_2} & \text{if } x = y. \end{cases} . \tag{47}$$

5 *ExpCM* with the preferences as free parameters

In most situations, the amino-acid preferences $\pi_{r,a}$ are experimentally measured. But in certain situations, we wish to treat these as free parameters that we optimize by maximum likelihood. There are two different implementations of how this is done, instantiated in the `ExpCM_fitprefs` and `ExpCM_fitprefs2` classes. These classes differ in how the preferences are represented as parameters, and so may have different optimization efficiencies.

First, we describe aspects general to both implementations, then we describe the details specific to each.

The $F_{r,xy}$ terms defined by Equation Frxy depend on $\pi_{r,a}$. The derivative is

$$\frac{\partial F_{r,xy}}{\partial \pi_{r,a}} = \begin{cases} \left(\delta_{a\mathcal{A}(y)} - \delta_{a\mathcal{A}(x)}\right) \frac{\omega\beta}{2\pi_{r,a}} & \text{if } \pi_{r,\mathcal{A}(x)} = \pi_{r,\mathcal{A}(y)}, \\ \left(\delta_{a\mathcal{A}(y)} - \delta_{a\mathcal{A}(x)}\right) \frac{\omega\beta}{\pi_{r,a}} \frac{\left(\pi_{r,\mathcal{A}(x)}/\pi_{r,\mathcal{A}(y)}\right)^\beta \left[\ln\left(\left(\frac{\pi_{r,\mathcal{A}(x)}}{\pi_{r,\mathcal{A}(y)}}\right)^\beta\right) - 1\right] + 1}{\left(1 - \left(\frac{\pi_{r,\mathcal{A}(x)}}{\pi_{r,\mathcal{A}(y)}}\right)^\beta\right)^2} & \text{if } \pi_{r,\mathcal{A}(x)} \neq \pi_{r,\mathcal{A}(y)}, \end{cases} \tag{48}$$

where the expressions when $\pi_{r,\mathcal{A}(x)} = \pi_{r,\mathcal{A}(y)}$ are derived from application of L'Hopital's rule, and δ_{ij} is the Kronecker delta.

Define

$$\tilde{F}_{r,xy} = \begin{cases} 0 & \text{if } \mathcal{A}\left(x\right) = \mathcal{A}\left(y\right), \\ \frac{\omega\beta}{2} & \text{if } \mathcal{A}\left(x\right) \neq \mathcal{A}\left(y\right) \text{ and } \pi_{r,\mathcal{A}(x)} = \pi_{r,\mathcal{A}(y)}, \\ \left(\omega\beta\right) \frac{\left(\pi_{r,\mathcal{A}(x)}/\pi_{r,\mathcal{A}(y)}\right)^\beta \left[\ln\left(\left(\frac{\pi_{r,\mathcal{A}(x)}}{\pi_{r,\mathcal{A}(y)}}\right)^\beta\right) - 1\right] + 1}{\left(1 - \left(\frac{\pi_{r,\mathcal{A}(x)}}{\pi_{r,\mathcal{A}(y)}}\right)^\beta\right)^2} & \text{if } \mathcal{A}\left(x\right) \neq \mathcal{A}\left(y\right) \text{ and } \pi_{r,\mathcal{A}(x)} \neq \pi_{r,\mathcal{A}(y)}, \end{cases} \tag{49}$$

so that

$$\frac{\partial F_{r,xy}}{\partial \pi_{r,a}} = \left(\delta_{a\mathcal{A}(y)} - \delta_{a\mathcal{A}(x)}\right) \frac{\tilde{F}_{r,xy}}{\pi_{r,a}}. \tag{50}$$

We also need to calculate the derivative of the stationary state $p_{r,x}$ given by Equation prx with respect to the preference. In this calculation, we simplify the algebra by taking advantage of the fact that for our fit preferences models, we always have $\beta = 1$ to simplify from the first to the second line below:

$$\frac{\partial p_{r,x}}{\partial \pi_{r,a}} = \frac{\partial}{\partial \pi_{r,x}} \left(\frac{q_x \left(\pi_{r,\mathcal{A}(x)}\right)^{\beta}}{\sum_z q_z \left(\pi_{r,\mathcal{A}(z)}\right)^{\beta}} \right) \tag{51}$$

$$= \frac{\partial}{\partial \pi_{r,x}} \left(\frac{q_x \pi_{r,\mathcal{A}(x)}}{\sum_z q_z \pi_{r,\mathcal{A}(z)}} \right) \tag{52}$$

$$= \frac{q_x \delta_{a\mathcal{A}(x)} \left(\sum_z q_z \pi_{r,\mathcal{A}(z)}\right) - q_x \pi_{r,\mathcal{A}(x)} \times \sum_z q_z \delta_{a\mathcal{A}(z)}}{\left(\sum_z q_z \pi_{r,\mathcal{A}(z)}\right)^2} \tag{53}$$

$$= \delta_{a\mathcal{A}(x)} \frac{p_{r,x}}{\pi_{r,a}} - p_{r,x} \sum_z \delta_{a\mathcal{A}(z)} \frac{p_{r,z}}{\pi_{r,a}}. \tag{54}$$

5.1 `ExpCM_fitprefs` implementation

We define a variable transformation from the 20 $\pi_{r,a}$ values at each site r (19 of these 20 values are unique since they sum to one). This transformation is analogous to that from ϕ to η above. Specifically, we number the 20 amino acids such that $a = 0$ means alanine, $a = 1$ means cysteine, and so on up to $a = 19$ meaning tyrosine.. We then define 19 free variables for each site r: $\zeta_{r,0}, \zeta_{r,1}, \ldots, \zeta_{r,18}$, all of which are constrained to value between zero and one. For notational convenience, we also define $\zeta_{r,19} = 0$, but not that $\zeta_{r,19}$ is **not** a free parameter as it is always zero.

We the define

$$\pi_{r,a} = \left(\prod_{i=0}^{a-1} \zeta_{r,i} \right) \left(1 - \zeta_{r,a}\right) \tag{55}$$

and conversely

$$\zeta_{r,a} = 1 - \pi_{r,a} / \left(\prod_{i=0}^{a-1} \zeta_{r,i} \right). \tag{56}$$

Note that setting $\zeta_{r,a} = \frac{19-a}{20-a}$ makes all the $\pi_{r,a}$ values equal to $\frac{1}{20}$.

We have

$$\frac{\partial \pi_{r,a}}{\partial \zeta_{r,i}} = \begin{cases} \frac{\pi_{r,a}}{\zeta_{r,i} - \delta_{ia}} & \text{if } i \le a, \\ 0 & \text{otherwise,} \end{cases} \tag{57}$$

where δ_{ij} is the Kronecker-delta.

We then have

$$\frac{\partial P_{r,xy}}{\partial \zeta_{r,i}} = Q_{xy} \sum_a \frac{\partial F_{r,xy}}{\partial \pi_{r,a}} \frac{\partial \pi_{r,a}}{\partial \zeta_{r,i}} = \begin{cases} 0 & \text{if } i > \mathcal{A}(x) \text{ and } i > \mathcal{A}(y) \text{ and } x \neq y, \\ \frac{Q_{xy} \tilde{F}_{r,xy}}{\zeta_{r,i} - \delta_{i\mathcal{A}(y)}} & \text{if } i > \mathcal{A}(x) \text{ and } i \le \mathcal{A}(y) \text{ and } x \neq y, \\ -\frac{Q_{xy} \tilde{F}_{r,xy}}{\zeta_{r,i} - \delta_{i\mathcal{A}(x)}} & \text{if } i \le \mathcal{A}(x) \text{ and } i > \mathcal{A}(y) \text{ and } x \neq y, \\ \frac{Q_{xy} \tilde{F}_{r,xy}}{\zeta_{r,i} - \delta_{i\mathcal{A}(y)}} - \frac{Q_{xy} \tilde{F}_{r,xy}}{\zeta_{r,i} - \delta_{i\mathcal{A}(x)}} & \text{if } i \le \mathcal{A}(x) \text{ and } i \le \mathcal{A}(y) \text{ and } x \neq y \\ -\sum\limits_{z \neq x} \frac{\partial P_{r,xy}}{\partial \zeta_{r,i}} & \text{if } x = y. \end{cases} \tag{58}$$

We also have:

$$\frac{\partial p_{r,x}}{\partial \zeta_{r,i}} = \sum_a \frac{\partial p_{r,x}}{\partial \pi_{r,a}} \frac{\partial \pi_{r,a}}{\partial \zeta_{r,i}} \tag{59}$$

$$= p_{r,x} \sum_{a \geq i} \frac{1}{\zeta_{r,i} - \delta_{ia}} \left(\delta_{a\mathcal{A}(x)} - \sum_z \delta_{a\mathcal{A}(z)} p_{r,z} \right). \tag{60}$$

5.2 `ExpCM_prefs2` implementation

For this implementation, we define a different variable transformation from the 20 $\pi_{r,a}$ values at each site r (19 of these 20 values are unique since they sum to one). We define 19 free variables for each site r: $\zeta_{r,0}, \zeta_{r,1}, \ldots, \zeta_{r,18}$, all of which are constrained to be greater than zero. For notational convenience, we also define $\zeta_{r,19} = 1$, but not that $\zeta_{r,19}$ is **not** a free parameter as it is always one.

We then define

$$\pi_{r,a} = \frac{\zeta_{r,a}}{\sum_j \zeta_{r,j}} \tag{61}$$

and conversely

$$\zeta_{r,a} = \frac{\pi_{r,a}}{\pi_{r,19}}. \tag{62}$$

We therefore have

$$\frac{\partial \pi_{r,a}}{\partial \zeta_{r,i}} = \frac{1}{\sum_j \zeta_{r,j}} \left(\delta_{ia} - \frac{\zeta_{r,a}}{\sum_j \zeta_{r,j}} \right) \tag{63}$$

$$= \frac{\pi_{r,a}}{\zeta_{r,a}} \left(\delta_{ia} - \pi_{r,a} \right) \tag{64}$$

where δ_{ij} is the Kronecker-delta.

We then have

$$\frac{\partial P_{r,xy}}{\partial \zeta_{r,i}} = Q_{xy} \sum_a \frac{\partial F_{r,xy}}{\partial \pi_{r,a}} \frac{\partial \pi_{r,a}}{\partial \zeta_{r,i}} \tag{65}$$

$$= Q_{xy} \tilde{F}_{r,xy} \sum_a \left(\delta_{a\mathcal{A}(y)} - \delta_{a\mathcal{A}(x)} \right) \frac{1}{\zeta_{r,a}} \left(\delta_{ia} - \pi_{r,a} \right) \tag{66}$$

$$= Q_{xy} \tilde{F}_{r,xy} \left[\left(\sum_a \left(\delta_{a\mathcal{A}(y)} - \delta_{a\mathcal{A}(x)} \right) \frac{\delta_{ia}}{\zeta_{r,a}} \right) - \left(\sum_a \left(\delta_{a\mathcal{A}(y)} - \delta_{a\mathcal{A}(x)} \right) \frac{\pi_{r,a}}{\zeta_{r,a}} \right) \right] \tag{67}$$

$$= Q_{xy} \tilde{F}_{r,xy} \left[\frac{\delta_{i\mathcal{A}(y)} - \delta_{i\mathcal{A}(x)}}{\zeta_{r,i}} - \left(\sum_a \left(\delta_{a\mathcal{A}(y)} - \delta_{a\mathcal{A}(x)} \right) \frac{\pi_{r,a}}{\zeta_{r,a}} \right) \right] \tag{68}$$

$$= Q_{xy} \tilde{F}_{r,xy} \left[\frac{\delta_{i\mathcal{A}(y)} - \delta_{i\mathcal{A}(x)}}{\zeta_{r,i}} - \left(\frac{1}{\sum_j \zeta_{r,j}} \sum_a \left(\delta_{a\mathcal{A}(y)} - \delta_{a\mathcal{A}(x)} \right) \right) \right] \tag{69}$$

$$= Q_{xy} \tilde{F}_{r,xy} \left[\frac{\delta_{i\mathcal{A}(y)} - \delta_{i\mathcal{A}(x)}}{\zeta_{r,i}} \right]. \tag{70}$$

We also have:

$$\begin{aligned}
\frac{\partial p_{r,x}}{\partial \zeta_{r,i}} &= \sum_a \frac{\partial p_{r,x}}{\partial \pi_{r,a}} \frac{\partial \pi_{r,a}}{\partial \zeta_{r,i}} &(71)\\
&= p_{r,x} \sum_a \frac{\delta_{ia} - \pi_{r,a}}{\zeta_{r,a}} \left(\delta_{a\mathcal{A}(x)} - \sum_z \delta_{a\mathcal{A}(z)} p_{r,z} \right) &(72)\\
&= p_{r,x} \left[\frac{1}{\zeta_{r,i}} \left(\delta_{i\mathcal{A}(x)} - \sum_z \delta_{i\mathcal{A}(z)} p_{r,z} \right) - \sum_a \frac{\pi_{r,a}}{\zeta_{r,a}} \left(\delta_{a\mathcal{A}(x)} - \sum_z \delta_{a\mathcal{A}(z)} p_{r,z} \right) \right] &(73)\\
&= p_{r,x} \left[\frac{1}{\zeta_{r,i}} \left(\delta_{i\mathcal{A}(x)} - \sum_z \delta_{i\mathcal{A}(z)} p_{r,z} \right) - \frac{\pi_{r,\mathcal{A}(x)}}{\zeta_{r,\mathcal{A}(x)}} + \sum_a \frac{\pi_{r,a}}{\zeta_{r,a}} \sum_z \delta_{a\mathcal{A}(z)} p_{r,z} \right] &(74)\\
&= p_{r,x} \left[\frac{1}{\zeta_{r,i}} \left(\delta_{i\mathcal{A}(x)} - \sum_z \delta_{i\mathcal{A}(z)} p_{r,z} \right) - \frac{1}{\sum_j \zeta_{r,j}} + \frac{1}{\sum_j \zeta_{r,j}} \sum_a \sum_z \delta_{a\mathcal{A}(z)} p_{r,z} \right] &(75)\\
&= \frac{p_{r,x}}{\zeta_{r,i}} \left(\delta_{i\mathcal{A}(x)} - \sum_z \delta_{i\mathcal{A}(z)} p_{r,z} \right). &(76)
\end{aligned}$$

6 Regularizing preferences for *ExpCM* with preferences as free parameters

When the preferences are free parameters, we typically want to regularize them to avoid fitting lots of values of one or zero. We do this by defining a regularizing prior over the preferences, and then maximizing the product of the likelihood and this regularizing prior (essentially, the *maximum a posteriori* estimate).

6.1 Inverse-quadratic prior

This is the prior used in [1] (note that the notation used here is slightly different than in that reference). Let $\pi_{r,a}$ be the preference that we are trying to optimize, and let $\theta_{r,a}$ be our prior estimate of $\pi_{r,a}$. Typically, this estimate is the original experimentally measured preference $\pi_{r,a}^{\mathrm{orig}}$ re-scaled by the optimized stringency parameter β, namely $\theta_{r,a} = \frac{\left(\pi_{r,a}^{\mathrm{orig}}\right)^\beta}{\sum_{a'} \left(\pi_{r,a'}^{\mathrm{orig}}\right)^\beta}$.

The prior is then

$$\Pr\left(\{\pi_{r,a}\} \mid \{\theta_{r,a}\}\right) = \prod_r \prod_a \left(\frac{1}{1 + C_1 \times \left(\pi_{r,a} - \theta_{r,a}\right)^2} \right)^{C_2}. \tag{77}$$

or

$$\ln\left[\Pr\left(\{\pi_{r,a}\} \mid \{\theta_{r,a}\}\right)\right] = -C_2 \sum_r \sum_a \ln\left(1 + C_1 \times \left(\pi_{r,a} - \theta_{r,a}\right)^2\right) \tag{78}$$

where C_1 and C_2 are parameters that specify how concentrated the prior is (larger values make the prior more strongly peaked at $\theta_{r,a}$).

The derivative is

$$\frac{\partial \ln \left[\Pr\left(\left\{\pi_{r,a}\right\} \mid \left\{\theta_{r,a}\right\}\right)\right]}{\partial \pi_{r,a}} = \frac{-2C_1C_2\left(\pi_{r,a} - \theta_{r,a}\right)}{1 + C_1 \times \left(\pi_{r,a} - \theta_{r,a}\right)^2}, \tag{79}$$

This prior can then be defined in terms of the transformation variable for the `ExpCM_fitprefs` or `ExpCM_fitprefs2` implementation:

6.1.1 `ExpCM_fitprefs` implementation

$$\begin{aligned}
\frac{\partial \ln \left[\Pr\left(\left\{\pi_{r,a}\right\} \mid \left\{\theta_{r,a}\right\}\right)\right]}{\partial \zeta_{r,i}} &= \sum_a \frac{\partial \ln \left[\Pr\left(\left\{\pi_{r,a}\right\} \mid \left\{\theta_{r,a}\right\}\right)\right]}{\partial \pi_{r,a}} \frac{\partial \pi_{r,a}}{\partial \zeta_{r,i}} && (80)\\
&= -2C_1C_2 \sum_{a \geq i} \frac{\left(\pi_{r,a} - \theta_{r,a}\right)}{1 + C_1 \times \left(\pi_{r,a} - \theta_{r,a}\right)^2} \frac{\pi_{r,a}}{\zeta_{r,i} - \delta_i a}. && (81)
\end{aligned}$$

6.1.2 `ExpCM_fitprefs2` implementation

$$\begin{aligned}
\frac{\partial \ln \left[\Pr\left(\left\{\pi_{r,a}\right\} \mid \left\{\theta_{r,a}\right\}\right)\right]}{\partial \zeta_{r,i}} &= \sum_a \frac{\partial \ln \left[\Pr\left(\left\{\pi_{r,a}\right\} \mid \left\{\theta_{r,a}\right\}\right)\right]}{\partial \pi_{r,a}} \frac{\partial \pi_{r,a}}{\partial \zeta_{r,i}} && (82)\\
&= -2C_1C_2 \sum_a \frac{\left(\pi_{r,a} - \theta_{r,a}\right)}{1 + C_1 \times \left(\pi_{r,a} - \theta_{r,a}\right)^2} \frac{\pi_{r,a}}{\zeta_{r,a}} \left(\delta_{ia} - \pi_{r,a}\right) && (83)\\
&= \frac{-2C_1C_2}{\sum_j \zeta_{r,j}} \sum_a \frac{\left(\pi_{r,a} - \theta_{r,a}\right)}{1 + C_1 \times \left(\pi_{r,a} - \theta_{r,a}\right)^2} \left(\delta_{ia} - \pi_{r,a}\right). && (84)
\end{aligned}$$

7 ***YNGKP_M0*** model

We consider the basic Goldman-Yang style *YNGKP_M0* substitution model defined in [2]. This model is **not** site-specific. P_{xy} is the substitution rate from codon x to codon y and is defined by

$$P_{xy} = \begin{cases} 0 & \text{if } x \text{ and } y \text{ differ by more than one nucleotide,} \\ \mu\omega\Phi_y & \text{if } x \text{ is converted to } y \text{ by a single-nucleotide transversion,} \\ \kappa\mu\omega\Phi_y & \text{if } x \text{ is converted to } y \text{ by a single-nucleotide transition,} \\ -\sum\limits_{z \neq x} P_{xz} & \text{if } x = y. \end{cases} \tag{85}$$

where κ is the transition-transversion ratio, Φ_y is the equilibrium frequency of codon y, ω is the gene-wide rate of non-synonymous change, and μ is the substitution rate. Typically Φ_y is determined empirically as described below, and κ and ω are optimized by maximum likelihood.

The derivatives are:

$$\frac{\partial P_{xy}}{\partial \kappa} = \begin{cases} \frac{P_{xy}}{\kappa} & \text{if } x \text{ is converted to } y \text{ by a transition of a nucleotide to } w, \\ 0 & \text{if } x \text{ and } y \text{ differ by something other than a single transition,} \\ -\sum\limits_{z \neq x} \frac{\partial P_{xz}}{\partial \kappa} & \text{if } x = y. \end{cases} \tag{86}$$

$$\frac{\partial P_{xy}}{\partial \omega} = \begin{cases} 0 & \text{if } \mathrm{A}\,(x) = \mathrm{A}\,(y) \text{ and } x \neq y \\ \frac{P_{xy}}{\omega} & \text{if } \mathrm{A}\,(x) \neq \mathrm{A}\,(y), \\ -\sum\limits_{z \neq x} \frac{\partial P_{xz}}{\partial \omega} & \text{if } x = y. \end{cases} \tag{87}$$

The stationary state of the substitution model defined by P_{xy} is

$$p_x = \Phi_x \tag{88}$$

The derivatives of the stationary state with respect to κ and ω are zero as these do not affect that state, so:

$$\frac{\partial p_x}{\partial \kappa} = \frac{\partial p_x}{\partial \omega} = 0 \tag{89}$$

We calculate the codon frequencies Φ_x from the observed nucleotide frequencies.

The original *F3X4* method calculated Φ_x directly from the empirical alignment frequencies. Specifically, let e^p_w be the empirical frequency of nucleotide w at codon position p. In the original *F3X4* method, $\Phi_x = e^1_{x_1} \times e^2_{x_2} \times e^3_{x_3}$. This method produces biased codon frequencies because the stop codon nucleotide composition is not taken into account.

To address this issue, we follow the *Corrected F3X4* (or *CF3X4*) method from [3]. The 12 nucleotide corrected nucleotide frequency parameters ϕ^p_w are estimated from the observed nucleotide frequencies by solving a set of 12 nonlinear equations:

$$e^1_w = \frac{\phi^1_w \times \left(1 - \sum\limits_{wyz \epsilon X} \phi^2_y \times \phi^3_z\right)}{1 - \sum\limits_{xyz \epsilon X} \phi^1_x \times \phi^2_y \times \phi^3_z} \tag{90}$$

$$e^2_w = \frac{\phi^2_w \times \left(1 - \sum\limits_{ywz \epsilon X} \phi^1_y \times \phi^3_z\right)}{1 - \sum\limits_{xyz \epsilon X} \phi^1_x \times \phi^2_y \times \phi^3_z} \tag{91}$$

$$e^3_w = \frac{\phi^3_w \times \left(1 - \sum\limits_{yzw \epsilon X} \phi^1_y \times \phi^2_z\right)}{1 - \sum\limits_{xyz \epsilon X} \phi^1_x \times \phi^2_y \times \phi^3_z} \tag{92}$$

$$\tag{93}$$

where $X = \{TAA, TAG, TGA\}$. We use the ϕ^p_w values determined in this way to compute $\Phi_x = \phi^1_{x_1} \times \phi^2_{x_2} \times \phi^3_{x_3}$.

8 Exponentials of the substitution matrix and derivatives

The definitions above can be used to define a set of matrices $\mathbf{P_r} = [P_{r,xy}]$ that give the rate of transition from codon x to y at site r. A key computation is to compute the probability of a

transition in some amount of elapsed time μt. These probabilities are given by

$$\mathbf{M_r}\left(\mu t\right) = e^{\mu t \mathbf{P_r}}. \tag{94}$$

In this section, we deal with how to compute $\mathbf{M_r}\left(\mu t\right)$ and its derivatives. Because $\mathbf{P_r}$ is reversible with stationary state given by the vector $\mathbf{p_r} = \left[p_{r,x}\right]$, then as described by [4], the matrix $\left[\operatorname{diag}\left(\mathbf{p_r}\right)\right]^{\frac{1}{2}} \mathbf{P_r} \left[\operatorname{diag}\left(\mathbf{p_r}\right)\right]^{\frac{-1}{2}}$ is symmetric.

We can use a numerical routine to compute the eigenvalues and orthonormal eigenvectors. Let $\mathbf{D_r}$ be a diagonal matrix with elements equal to the eigenvalues, let $\mathbf{B_r}$ be the matrix whose columns are the right orthonormal eigenvectors (in the same order as the eigenvalues), and note that $\mathbf{B_r}^{-1} = \mathbf{B_r}^T$. Then we have $\left[\operatorname{diag}\left(\mathbf{p_r}\right)\right]^{\frac{1}{2}} \mathbf{P_r} \left[\operatorname{diag}\left(\mathbf{p_r}\right)\right]^{\frac{-1}{2}} = \mathbf{B_r} \mathbf{D_r} \mathbf{B_r}^T$ or equivalently

$$\mathbf{P_r} = \mathbf{A_r} \mathbf{D_r} \mathbf{A_r}^{-1} \tag{95}$$

where

$$\mathbf{A_r} = \left[\operatorname{diag}\left(\mathbf{p_r}\right)\right]^{\frac{-1}{2}} \mathbf{B_r} \tag{96}$$

and

$$\mathbf{A_r}^{-1} = \mathbf{B_r}^T \left[\operatorname{diag}\left(\mathbf{p_r}\right)\right]^{\frac{1}{2}}. \tag{97}$$

The matrix exponentials are then easily calculated as

$$\mathbf{M_r}\left(\mu t\right) = e^{\mu t \mathbf{P_r}} = \mathbf{A_r} e^{\mu t \mathbf{D_r}} \mathbf{A_r}^{-1}. \tag{98}$$

We also want to calculate the derivatives of $\mathbf{M_r}\left(\mu t\right)$ with respect to the other parameters on which $P_{r,xy}$ depends (e.g., β, η_i, κ, and ω).

According to [5] (see also [6, 7]), the derivative with respect to some parameter z is given by

$$\frac{\partial \mathbf{M_r}\left(\mu t\right)}{\partial z} = \mathbf{A_r} \mathbf{V_{r,z}} \mathbf{A_r}^{-1} \tag{99}$$

where the elements of $\mathbf{V_{r,z}}$ are

$$V_{xy}^{r,z} = \begin{cases} B_{xy}^{r,z} \frac{\exp\left(\mu t D_{xx}^r\right) - \exp\left(\mu t D_{yy}^r\right)}{D_{xx}^r - D_{yy}^r} & \text{if } x \neq y \text{ and } D_{xx}^r \neq D_{yy}^r, \\ B_{xy}^{r,z} \mu t \exp\left(\mu t D_{xx}^r\right) & \text{if } x \neq y \text{ and } D_{xx}^r = D_{yy}^r, \\ B_{xx}^{r,z} \mu t \exp\left(\mu t D_{xx}^r\right) & \text{if } x = y, \end{cases} \tag{100}$$

where D_{xx}^r and D_{yy}^r are the diagonal elements of $\mathbf{D_r}$, and $B_{xy}^{r,z}$ are the elements of the matrix $\mathbf{B_{r,z}}$ defined by

$$\mathbf{B_{r,z}} = \mathbf{A_r}^{-1} \frac{\partial \mathbf{P_r}}{\partial z} \mathbf{A_r}. \tag{101}$$

9 Scaling the branch lengths with a mutation rate

The aforementioned section defines the substitution probabilities in terms of μt (e.g., Eq. Mr). Here μ is a substitution rate, and t is the branch length. If we are freely optimizing all branch

lengths, then we just set $\mu = 1$ so that $\mu t = t$, and then μ effectively drops out. However, if we have fixed the branch lengths are **not** optimizing them, then we might want to include a parameter μ that effectively re-scales all the fixed branch lengths by a constant. In this case, μ also becomes a free parameter of the model, and we want to compute the derivative of $\mathbf{M_r}(\mu t)$ with respect to μ. This is straightforward:

$$\frac{\partial \mathbf{M_r}(\mu t)}{\partial \mu} = t\mathbf{P_r} e^{\mu t \mathbf{P_r}} = t\mathbf{P_r}\mathbf{M_r}(\mu t). \tag{102}$$

10 Calculating the likelihood and derivatives on a tree

Above we describe computing the transition probabilities as a function of branch length. Here we consider how to use those computations to compute the actual likelihoods on a tree.

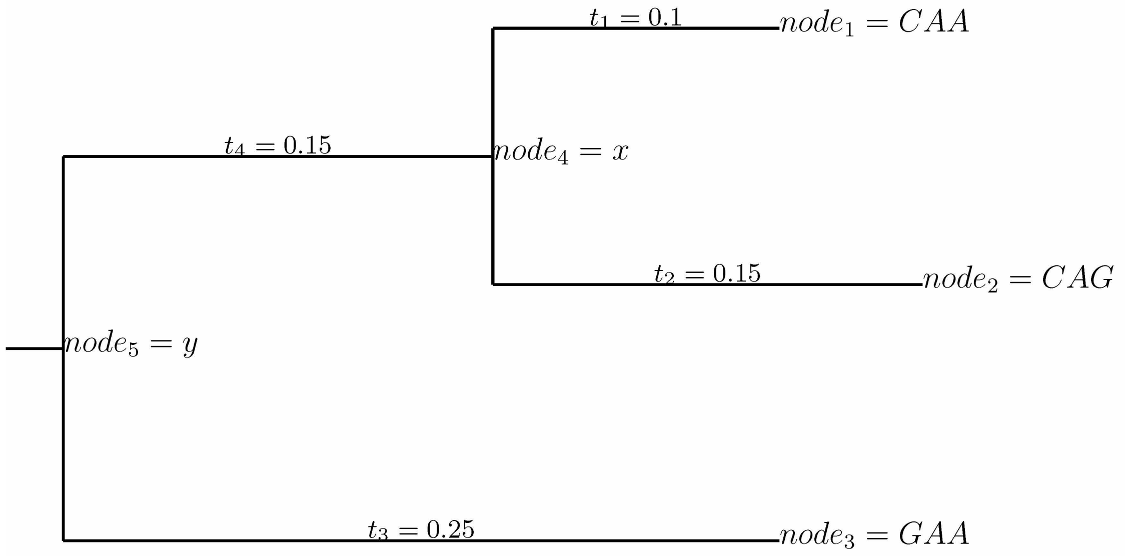

Figure 1: The tree used in the example calculation below.

We begin by computing the likelihood of the alignment at a specific site. Let $\mathcal{S}_r$ denote the set of aligned codons at site r, let $\mathcal{T}$ by the phylogenetic tree with branch lengths specified, and let $\mathbf{P_r}$ be the transition matrix at site r defined above. Then the likelihood at site r is $\Pr(\mathcal{S}_r \mid \mathcal{T}, \mathbf{P_r})$. For the example tree above, we can use the pruning algorithm [8] to write

$$\Pr(\mathcal{S}_r \mid \mathcal{T}, \mathbf{P_r}) = \sum_y p_{r,y} M_{r,yGAA}(t_3) \left[\sum_x M_{r,yx}(t_4) M_{r,xCAA}(t_1) M_{r,xCAG}(t_2)\right]. \tag{103}$$

Let n denote a node on a tree, let t_n denote the length of the branch leading to node n, and let $\lceil_1(n)$ and $\lceil_1(n)$ denote the right and left descendents of node n for all non-terminal nodes. Then

define the *partial conditional likelihood* of the subtree rooted at n as:

$$L_{r,n}\left(x\right)=\begin{cases}\delta_{x\mathcal{S}_{r,n}} & \text{if } n \text{ is a tip node with codon } \mathcal{S}_{r,n} \text{ at site } r,\\ 1 & \text{if } n \text{ is a tip node with a gap at site } r,\\ \left[\sum_y M_{r,xy}\left(t_{\lceil_1(n)}\right)L_{r,\lceil_1(n)}\left(y\right)\right]\left[\sum_y M_{r,xy}\left(t_{\lceil_2(n)}\right)L_{r,\lceil_2(n)}\left(y\right)\right] & \text{otherwise.}\end{cases} \quad ([illegible]$$

where δ_{xy} is the Kronecker delta. So for instance in the example tree above, $L_{r,n_4}\left(x\right)=M_{r,xCAA}\left(t_1\right)M_{r,xCAG}\left(t_2\right)$, and $L_{r,n_5}\left(y\right)=M_{r,yGAA}\sum_x M_{r,yx}\left(t_4\right)L_{r,n_4}\left(x\right)$.

Using this definition, we have

$$\Pr\left(\mathcal{S}_r \mid \mathcal{T},\mathbf{P_r}\right)=\sum_x p_{r,x}L_{r,n_{\text{root}}}\left(x\right) \tag{105}$$

where n_{root} is the root node of tree $\mathcal{T}$; $n_{\text{root}}=n_5$ in the example tree above.

In practice, we usually work with the log likelihoods (always using natural logarithms). The total likelihood is the sum of the log likelihoods for each site:

$$\ln\left[\Pr\left(\mathcal{S}\mid\mathcal{T},\left\{\mathbf{P_r}\right\}\right)\right]=\sum_r \ln\left[\Pr\left(\mathcal{S}_r\mid\mathcal{T},\mathbf{P_r}\right)\right]. \tag{106}$$

We next consider how to compute the derivatives with respect to some model parameter. Let α denote the model parameter in question, and assume that we have already determined $\frac{M_{r,xy}(t)}{\partial\alpha}$. By the chain rule, we have

$$\frac{\partial L_{r,n}\left(x\right)}{\partial\alpha}=\begin{cases}0 & \text{if } n \text{ is a tip [illegible]}\\ \left[\sum_y \frac{\partial M_{r,xy}\left(t_{\lceil_1(n)}\right)}{\partial\alpha}L_{r,\lceil_1(n)}\left(y\right)+M_{r,xy}\left(t_{\lceil_1(n)}\right)\frac{\partial L_{r,\lceil_1(n)}(y)}{\partial\alpha}\right]\left[\sum_y M_{r,xy}\left(t_{\lceil_2(n)}\right)L_{r,\lceil_2(n)}\left(y\right)\right] & \text{otherwise.}\\ +\left[\sum_y M_{r,xy}\left(t_{\lceil_1(n)}\right)L_{r,\lceil_1(n)}\left(y\right)\right]\left[\sum_y \frac{\partial M_{r,xy}\left(t_{\lceil_2(n)}\right)}{\partial\alpha}L_{r,\lceil_2(n)}\left(y\right)+M_{r,xy}\left(t_{\lceil_2(n)}\right)\frac{\partial L_{r,\lceil_2(n)}(y)}{\partial\alpha}\right] & \end{cases}$$

The derivative of the likelihood at the site is then

$$\frac{\partial\Pr\left(\mathcal{S}_r\mid\mathcal{T},\mathbf{P_r}\right)}{\partial\alpha}=\sum_x\left(\frac{\partial p_{r,x}}{\partial\alpha}L_{r,n_{\text{root}}}\left(x\right)+p_{r,x}\frac{\partial L_{r,n_{\text{root}}}\left(x\right)}{\partial\alpha}\right) \tag{108}$$

and the derivative of the log likelihood at the site is

$$\frac{\partial\ln\left[\Pr\left(\mathcal{S}_r\mid\mathcal{T},\mathbf{P_r}\right)\right]}{\partial\alpha}=\frac{\sum_x\left(\frac{\partial p_{r,x}}{\partial\alpha}L_{r,n_{\text{root}}}\left(x\right)+p_{r,x}\frac{\partial L_{r,n_{\text{root}}}(x)}{\partial\alpha}\right)}{\Pr\left(\mathcal{S}_r\mid\mathcal{T},\mathbf{P_r}\right)}. \tag{109}$$

The derivative of the overall log likelihood is

$$\frac{\partial\ln\left[\Pr\left(\mathcal{S}\mid\mathcal{T},\left\{\mathbf{P_r}\right\}\right)\right]}{\partial\alpha}=\sum_r\frac{\partial\ln\left[\Pr\left(\mathcal{S}_r\mid\mathcal{T},\mathbf{P_r}\right)\right]}{\partial\alpha}. \tag{110}$$

11 Scaling to avoid numerical underflow

For larger trees, there can be numerical underflow due to multiplication of lots of small numbers when computing the likelihoods. This issue, and how it can be solved by re-scaling the likelihoods during the calculation, is discussed on page 426 of [9].

Let $L_{r,n}(x)$ be the partial conditional likelihood at node n of codon x at site r as defined above. These partial conditional likelihoods can get very small as we move up the tree towards the root, as they are recursively defined as the products of very small numbers. For the scaling to avoid underflow, we define the scaled partial condition likelilhood as

$$\tilde{L}_{r,n}(x) = \frac{L_{r,n}(x)}{U_{r,n} \times \prod\limits_{k<n} U_{r,k}} \tag{111}$$

where we use $k < n$ to indicate all nodes k that are descendants of n, and where

$$U_{r,n} = \begin{cases} 1 & \text{if } n \text{ is divisible by } K, \\ \max_x \left[L_{r,n}(x) \times \prod\limits_{k<n} U_{r,k}\right] & \text{otherwise} \end{cases} \tag{112}$$

where K is the frequency with which we re-scale the likelihoods. A reasonable value of K might be 5 or 10. Effectively, this means that every K nodes we are re-scaling so that the largest partial conditional likelihood is one.

With this re-scaling, the total likelihood at site r is then

$$\Pr(\mathcal{S}_r \mid \mathcal{T}, \mathbf{P_r}) = \left(\sum_x p_{r,x} \tilde{L}_{r,n_{\text{root}}}(x)\right) \times \left(\prod_n U_{r,n}\right) \tag{113}$$

and the total log likelihood at site r is

$$\ln\left[\Pr(\mathcal{S}_r \mid \mathcal{T}, \mathbf{P_r})\right] = \ln\left(\sum_x p_{r,x} \tilde{L}_{r,n_{\text{root}}}(x)\right) + \sum_n \ln(U_{r,n}). \tag{114}$$

The derivative is then

$$\begin{aligned}
\frac{\partial \ln\left[\Pr(\mathcal{S}_r \mid \mathcal{T}, \mathbf{P_r})\right]}{\partial \alpha} &= \frac{\frac{\partial}{\partial \alpha}\left[\left(\sum\limits_x p_{r,x} \tilde{L}_{r,n_{\text{root}}}(x)\right) \times \left(\prod\limits_n U_{r,n}\right)\right]}{\left(\sum\limits_x p_{r,x} \tilde{L}_{r,n_{\text{root}}}(x)\right) \times \left(\prod\limits_n U_{r,n}\right)} &(115)\\
&= \frac{\left(\sum\limits_x \left[\frac{\partial p_{r,x}}{\partial \alpha} \tilde{L}_{r,n_{\text{root}}}(x) + p_{r,x} \frac{\partial \tilde{L}_{r,n_{\text{root}}}(x)}{\partial \alpha}\right]\right) \times \left(\prod\limits_n U_{r,n}\right) + \left(\sum\limits_x p_{r,x} \tilde{L}_{r,n_{\text{root}}}(x)\right) \times \frac{\partial\left(\prod\limits_n U_{r,n}\right)}{\partial \alpha}}{\left(\sum\limits_x p_{r,x} \tilde{L}_{r,n_{\text{root}}}(x)\right) \times \left(\prod\limits_n U_{r,n}\right)} &(116)\\
&= \frac{\sum_x \left(\frac{\partial p_{r,x}}{\partial \alpha} \tilde{L}_{r,n_{\text{root}}}(x) + p_{r,x} \frac{\partial \tilde{L}_{r,n_{\text{root}}}(x)}{\partial \alpha}\right)}{\sum\limits_x p_{r,x} \tilde{L}_{r,n_{\text{root}}}(x)} + \frac{\frac{\partial\left(\prod\limits_n U_{r,n}\right)}{\partial \alpha}}{\prod\limits_n U_{r,n}}. &(117)
\end{aligned}$$

For reasons that are not immediately obvious to me but are clearly verified by numerical testing, this last term of $\frac{\frac{\partial\left(\prod_n U_{r,n}\right)}{\partial\alpha}}{\prod_n U_{r,n}}$ is zero, and so

$$\frac{\partial \ln\left[\Pr\left(\mathcal{S}_r \mid \mathcal{T}, \mathbf{P_r}\right)\right]}{\partial\alpha} = \frac{\sum_x \left(\frac{\partial p_{r,x}}{\partial\alpha}\tilde{L}_{r,n_{\text{root}}}\left(x\right) + p_{r,x}\frac{\partial\tilde{L}_{r,n_{\text{root}}}(x)}{\partial\alpha}\right)}{\sum_x p_{r,x}\tilde{L}_{r,n_{\text{root}}}\left(x\right)}. \tag{118}$$

In practice, we work with the $\tilde{L}_{r,n}\left(x\right)$ values, and then apply the correction of adding $\sum_n \ln\left(U_r, n\right)$ at the end.

12 Units of tree branch lengths

When we optimize with the $P_{r,xy}$ substitution matrices described above, the resulting branch lengths are **not** in units of substitutions per site. Therefore, for tree input / output, we re-scale the branch lengths so that they are in units of substitution per site.

In a single unit of time, the probability that if site r is initially x, then it will undergo a substitution to some other codon y is $\sum_{y\neq x} P_{r,xy} = -P_{r,xx}$. Since the equilibrium probability that site r is x is $p_{r,x}$, then the probability that site r undergoes a substitution in a unit of time is $-\mu\sum_x p_{r,x}P_{r,xx}$. So averaging over all L sites, the probability that the average site will undergo a substitution in a unit of time is $-\frac{\mu}{L}\sum_{r=1}^{L}\sum_x p_{r,x}P_{r,xx}$.

Therefore, if we optimize the branch lengths t_b and the model parameters in $P_{r,xy}$, and then at the end re-scale the branch lengths to $t_b' = t_b \times \frac{-\mu}{L}\sum_{r=1}^{L}\sum_x p_{r,x}P_{r,xx}$ then the re-scaled branch lengths t_b are in units of substitutions per sites. Therefore, for input and output to `phydms`, we assume that input branch lengths are already in units of substitutions per site, and scale them from t_b' to t_b. Optimization is performed on t_b, and then for output we re-scale the optimized branch lengths from t_b to t_b'.

13 Models with gamma-distributed ω

The models described above fit a single ω value. We can also fit a distribution of ω values across sites. For instance, when this is done for the *YNGKP* models, we get the *YNGKP_M5* model described in [2].

Specifically, let the ω values be drawn from K discrete categories with omega values $\omega_0, \omega_2, \ldots, \omega_{K-1}$, and give equal weight to each category. Then the overall likelihood at site r is

$$\Pr\left(\mathcal{S}_r \mid \mathcal{T}, \mathbf{P_r}\right) = \frac{1}{K}\sum_{k=0}^{K-1}\Pr\left(\mathcal{S}_r \mid \mathcal{T}, \mathbf{P_r}_{\omega=\omega_k}\right) \tag{119}$$

and the derivative with respect to model parameter λ is simply

$$\frac{\partial\Pr\left(\mathcal{S}_r \mid \mathcal{T}, \mathbf{P_r}\right)}{\partial\lambda} = \frac{1}{K}\sum_{k=0}^{K-1}\frac{\partial\Pr\left(\mathcal{S}_r \mid \mathcal{T}, \mathbf{P_r}_{\omega=\omega_k}\right)}{\partial\lambda}. \tag{120}$$

The different ω_k values are drawn from the means of a gamma-distribution discretized into K categories as described by [10]. Specifically, this gamma distribution is described by a shape parameter α_ω and an inverse scale parameter β_ω such that the probability density function of a continuous ω is given by

$$g(\omega; \alpha_\omega, \beta_\omega) = \frac{(\beta_\alpha)^{\alpha_\omega} e^{-\beta_\omega \omega} \omega^{\alpha_\omega - 1}}{\Gamma(\alpha_\omega)}. \tag{121}$$

This function can be evaluated by `scipy.stats.gamma.pdf(omega, alpha_omega, scale=1.0 / beta_omega)`. Note also that the mean of this distribution is $\frac{\alpha_\omega}{\beta_\omega}$ and the variance is $\frac{\alpha_\omega}{(\beta_\omega)^2}$.

The lower and upper boundaries of the interval for each category k are

$$\omega_{k,\text{lower}} = Q_\Gamma\left(\frac{k}{K}; \alpha_\omega, \beta_\omega\right) \tag{122}$$

$$\omega_{k,\text{upper}} = Q_\Gamma\left(\frac{k+1}{K}; \alpha_\omega, \beta_\omega\right) \tag{123}$$

where Q_Γ is the quantile function (or percent-point function) of the gamma distribution. This function can be evaluated by `scipy.stats.gamma.ppf(k / K, alpha_omega, scale=1.0 / beta_omega)`.

The mean for each category k is

$$\omega_k = \frac{\alpha_\omega K}{\beta_\omega}\left[\gamma\left(\omega_{k,\text{upper}}\beta_\omega, \alpha_\omega + 1\right) - \gamma\left(\omega_{k,\text{lower}}\beta_\omega, \alpha_\omega + 1\right)\right] \tag{124}$$

where γ is the lower-incomplete gamma function and can be evaluated by `scipy.special.gammainc(alpha_omega + 1, omega_k_upper * beta_omega)`.

Note that ω_k is not actually a free parameter, as it is determined by α_ω and β_ω. The derivative of the log likelihood at site r with respect to these parameters is simply

$$\frac{\partial \Pr\left(\mathcal{S}_r \mid \mathcal{T}, \mathbf{P_r}\right)}{\partial \alpha_\omega} = \frac{1}{K}\sum_{k=0}^{K-1} \frac{\partial \omega_k}{\partial \omega_\alpha} \frac{\partial \Pr\left(\mathcal{S}_r \mid \mathcal{T}, \mathbf{P}_{\mathbf{r}\omega=\omega_k}\right)}{\partial \omega_k} \tag{125}$$

$$\frac{\partial \Pr\left(\mathcal{S}_r \mid \mathcal{T}, \mathbf{P_r}\right)}{\partial \beta_\omega} = \frac{1}{K}\sum_{k=0}^{K-1} \frac{\partial \omega_k}{\partial \omega_\beta} \frac{\partial \Pr\left(\mathcal{S}_r \mid \mathcal{T}, \mathbf{P}_{\mathbf{r}\omega=\omega_k}\right)}{\partial \omega_k}. \tag{126}$$

The derivatives $\frac{\partial \omega_k}{\partial \omega_\alpha}$ and $\frac{\partial \omega_k}{\partial \omega_\beta}$ are computed numerically using the finite-difference method.

14 Derivatives with respect to branch lengths

The section above describes how to compute the derivatives with respect to paramters (e.g., model parameters) that affect all parts of the tree. In many cases, we may want to optimize individual branch lengths rather than the overall mutation rate μ. In this case, we need to compute the derivatives with respect to the branch lengths. This is somewhat simpler for each individual branch length, since each individual branch length only affects part of the tree.

Specifically, for each internal node n,

$$\frac{\partial L_{r,n}(x)}{\partial t_{\lceil_1(n)}} = \frac{\partial}{\partial t_{\lceil_1(n)}}\left(\left[\sum_y M_{r,xy}\left(t_{\lceil_1(n)}\right) L_{r,\lceil_1(n)}(y)\right]\left[\sum_y M_{r,xy}\left(t_{\lceil_2(n)}\right) L_{r,\lceil_2(n)}(y)\right]\right) \quad (127)$$

$$= \left[\sum_y \frac{\partial M_{r,xy}\left(t_{\lceil_1(n)}\right)}{\partial t_{\lceil_1(n)}} L_{r,\lceil_1(n)}(y)\right]\left[\sum_y M_{r,xy}\left(t_{\lceil_2(n)}\right) L_{r,\lceil_2(n)}(y)\right] \quad (128)$$

where

$$\frac{\partial M_{r,xy}(t)}{\partial t} = \mu \mathbf{P_r} e^{\mu t \mathbf{P_r}} = \mu \mathbf{P_r} \mathbf{M_r}(\mu t). \quad (129)$$

Therefore, for every node n with descendents n_1 and n_2:

$$\frac{\partial L_{r,n}(x)}{\partial t_{n'}} = \begin{cases} 0 & \text{if } n' \text{ is not a descendent of } n \\ \left[\sum_y \frac{\partial M_{r,xy}(t_{n'})}{\partial t_{n'}} L_{r,n'}(y)\right]\left[\sum_y M_{r,xy}(t_{n_2}) L_{r,n_2}(y)\right] & \text{if } n_1 \text{ is } n' \\ \left[\sum_y M_{r,xy}(t_{n_1}) \frac{\partial L_{r,n_1}(y)}{\partial t_{n'}}\right]\left[\sum_y M_{r,xy}(t_{n_2}) L_{r,n_2}(y)\right] & \text{if } n' \text{ is descendent of } n_1 \end{cases} \quad (130)$$

and

$$\frac{\partial \Pr(\mathcal{S}_r \mid \mathcal{T}, \mathbf{P_r})}{\partial t_n} = \frac{\partial L_{r,n_{\text{root}}}(x)}{\partial t_n} \times p_{r,x}. \quad (131)$$

15 Optimization

The actual optimization is performed with the optimizer `scipy.optimize.minimize(method='L-BFGS-B')`. The approach is to first optimize all the model parameters along with branch-scaling parameter μ, then to optimize all the branch lengths, and to continue to repeat until any optimization step fails to lead to substantial further improvement in likelihood.

During the branch-length optimization, all branch lengths are updated simultaneously. This appears to be the minority approach in phylogenetics (most software does one branch length at a time), but reportedly some software does use simultaneous branch-length optimization (see table on page 18 of [4]).

References

[1] Jesse D Bloom. Identification of positive selection in genes is greatly improved by using experimentally informed site-specific models. *Biology Direct*, 12:1, 2017.

[2] Ziheng Yang, Rasmus Nielsen, Nick Goldman, and Anne-Mette Krabbe Pedersen. Codon-substitution models for heterogeneous selection pressure at amino acid sites. *Genetics*, 155(1):431–449, 2000.

[3] Sergei Kosakovsky Pond, Wayne Delport, Spencer V Muse, and Konrad Scheffler. Correcting the bias of empirical frequency parameter estimators in codon models. *PLoS One*, 5(7):e11230, 2010.

[4] David Bryant, Nicolas Galtier, and Marie-Anne Poursat. Likelihood calculation in molecular phylogenetics. *Mathematics of evolution and phylogeny*, pages 33–62, 2005.

[5] J. D. Kalbeisch and J. F. Lawless. The analysis of panel data under a Markov assumption. *Journal of the American Statistical Association*, 80:863–871, 1985.

[6] Toby Kenney, Hong Gu, et al. Hessian calculation for phylogenetic likelihood based on the pruning algorithm and its applications. *Statistical applications in genetics and molecular biology*, 11(4):1–46, 2012.

[7] Jesse D Bloom, Jagannath S Nayak, and David Baltimore. A computational-experimental approach identifies mutations that enhance surface expression of an oseltamivir-resistant influenza neuraminidase. *PloS One*, 6(7):e22201, 2011.

[8] J. Felsenstein. Evolutionary trees from DNA sequences: a maximum likelihood approach. *J. Mol. Evol.*, 17:368–376, 1981.

[9] Ziheng Yang. Maximum likelihood estimation on large phylogenies and analysis of adaptive evolution in human influenza virus a. *Journal of molecular evolution*, 51(5):423–432, 2000.

[10] Ziheng Yang. Maximum likelihood phylogenetic estimation from dna sequences with variable rates over sites: approximate methods. *J. Mol. Evol.*, 39(3):306–314, 1994.
